# Multi-d orbital and lattice distortion synergism boosting alkaline OER activity of carbon-supported IrFeCoNiMn high-entropy nanoparticles

**DOI:** 10.1039/d6ra06296b

**Published:** 2026-08-21

**Authors:** Xiaolong Ma, Yifan Huang, Qin Wang, Xinger Wang, Xin Liang, Gang Hu, Biao Qin

**Affiliations:** a School of Chemistry and Chemical Engineering, Zunyi Normal University Zunyi Guizhou 563006 China shawnma1990@163.com

## Abstract

To address the sluggish kinetics and poor long-term stability of conventional alkaline oxygen evolution electrocatalysts, a facile one-pot oil-phase synthetic strategy is developed to modulate the elemental stoichiometry and nanoparticle size of carbon-supported IrFeCoNiMn high-entropy alloys, which enables quantitative decoupling of geometric activity enhancements originating from lattice distortion and electronic promotions derived from well-established multi-d orbital interactions for alkaline OER catalysis. XRD, TEM, STEM mapping and ICP-OES characterisation methods verify a monophase fcc solid-solution structure with a homogeneous atomic distribution of five metals and an average particle size of 5.29 nm. In 1 M KOH electrolyte, the catalyst delivers superior OER activity: an ultralow overpotential of 241 mV at 10 mA cm^−2^ and a small Tafel slope of 46.15 mV dec^−1^, with its TOF at 300 mV overpotential, 53.95 times higher than commercial IrO_2_. Only a slight performance attenuation is observed after 5000 CV cycles and 80 h galvanostatic electrolysis at 10 mA cm^−2^, and post-characterization confirms intact crystal frameworks and trivial metal leaching under this test condition. High-resolution XPS and valence band spectra validate spontaneous charge transfer from 3d transition metals to 5d Ir, generating strong d–d orbital hybridisation and tuning the d-band centre to −4.155 eV. Such electronic rearrangement optimises intermediate adsorption and reduces the energy barrier of the rate-determining step. This work clarifies the synergistic catalytic mechanism of lattice distortion and multi-d orbital coupling, providing a feasible structural design strategy for low-noble OER catalysts with promising stability under medium-term alkaline electrolysis conditions.

## Abstract 2

Carbon‑supported IrFeCoNiMn high‑entropy alloys with lattice distortion and multi‑d‑orbital hybridization modulate electronic structure for efficient alkaline OER, delivering low overpotential and excellent long‑term galvanostatic stability.

## Graphical abstract

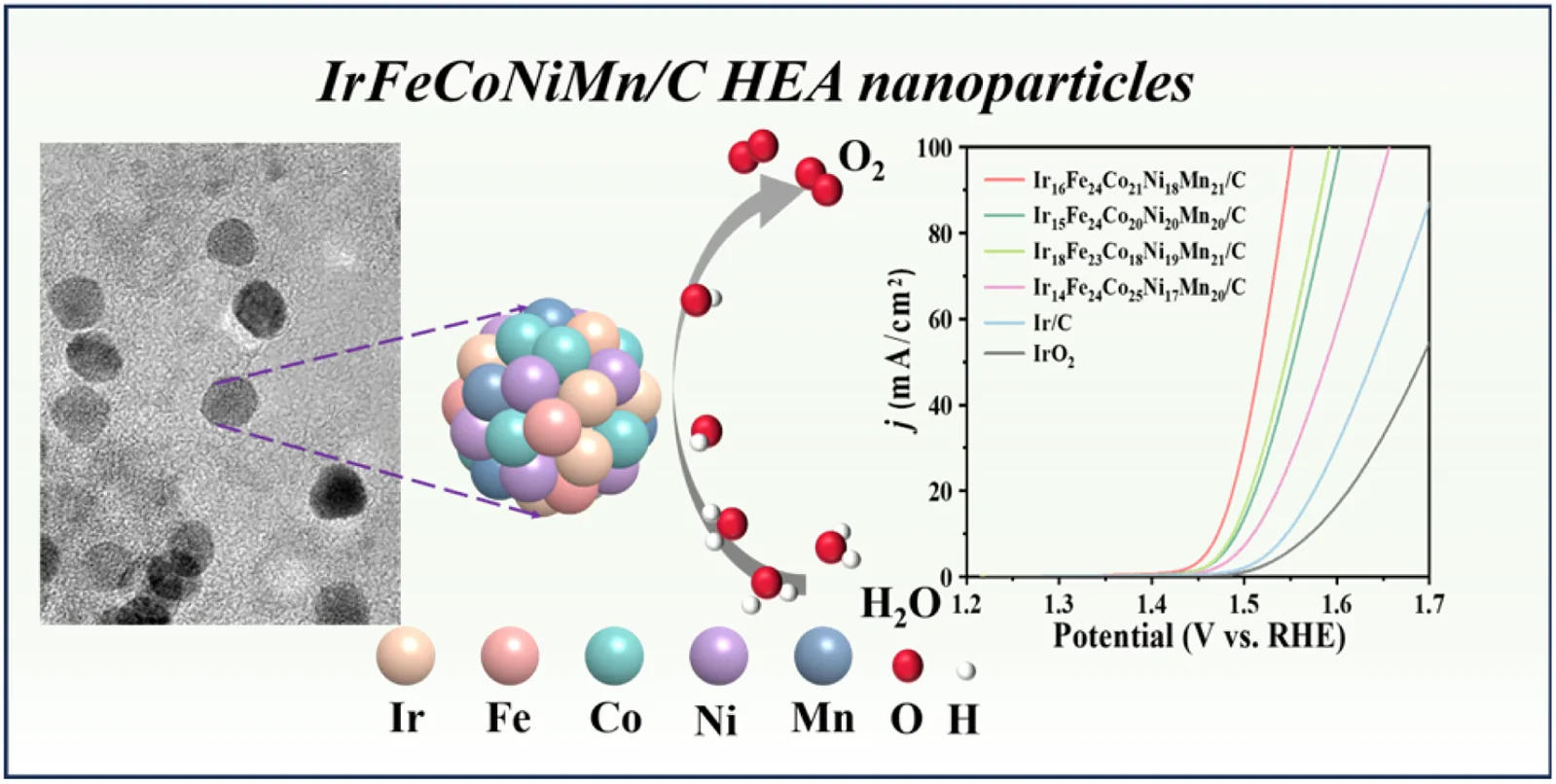

## Introduction

Fossil fuels have long underpinned global energy provision, yet their overexploitation and combustion have given rise to two intertwined crises: aggravated supply-demand imbalances in oil and gas coupled with elevated energy security risks, as well as enormous CO_2_ discharges that induce a chain of environmental disasters including global warming, glacial retreat, extreme climatic events, and the pervasive deterioration of terrestrial and marine ecosystems.^1,2^ These issues severely impede global low-carbon transitions and sustainable development.^3^ Hydrogen stands out as an ideal carbon-neutral alternative to fossil fuels, boasting high energy density, zero full-life-cycle carbon emissions and water as its sole oxidation product.^4,5^ Green hydrogen produced *via* renewable energy-driven water electrolysis eliminates fossil fuel reliance and greenhouse gas discharge, emerging as the dominant route for large-scale clean hydrogen manufacture.^6,7^ Water electrolysis relies on paired cathodic hydrogen evolution (HER) and anodic oxygen evolution (OER).^8,9^ Alkaline electrolytes are preferable to acidic counterparts for industrial deployment, as they tolerate low-cost non-precious metal catalysts, lower electrolyzer capital and operational costs, and improve catalyst anti-oxidation durability.^9,10^ Nevertheless, OER proceeds *via* an intricate four-electron proton-coupled transfer pathway.^11,12^ Variable adsorption/desorption barriers of key oxygen intermediates (*OH, *O, *OOH) lead to sluggish intrinsic kinetics, demanding large overpotentials and restricting the overall energy efficiency of electrolysis.^13,14^ Developing high-performance OER electrocatalysts with low overpotential, rapid kinetics and outstanding longevity is therefore critical to advancing industrial green hydrogen production. Commercial OER catalysts are dominated by IrO_2_ and RuO_2_, yet the extreme scarcity of Ir and Ru drives prohibitive material costs.^15,16^ Furthermore, prolonged harsh oxidative electrolysis induces severe noble metal leaching and structural collapse, resulting in rapid activity decay and hindering large-scale implementation.^17,18^ To resolve these limitations, developing low-noble or noble-alloyed electrocatalysts with simultaneously enhanced intrinsic activity and electrochemical stability has become a leading research focus in hydrogen electrocatalysis.

High-entropy alloys (HEAs) possess four unique inherent properties: high-entropy, lattice distortion, sluggish diffusion and cocktail effects.^19,20^ Multi-metal synergism enables precise tuning of d-orbital electronic configurations and balanced binding of OER intermediates, offering an innovative design strategy for alkaline OER catalysts.^10,15,21^ Single-component 3d high-entropy alloys exhibit sluggish catalytic kinetics: their contracted d-orbitals and insufficient valence electrons generate excessively strong, irreversible binding affinity for oxygen reaction intermediates, hindering interfacial electron transfer and accelerating passivation of active sites—both of which degrade catalytic activity and cycling durability.^22^ In contrast, 4d (Mo, Ru) and 5d noble metals (Ir, Pt) feature expanded d-orbitals and abundant valence electrons.^10,23,24^ The relativistic effect of 5d metals flexibly modulates electron cloud distribution and orbital hybridization strength, greatly accelerating interfacial charge transfer and corrosion resistance.^25,26^ Integrating 3d transition metals (Fe, Co, Ni, Mn) with 5d Ir to construct multi-d-orbital HEAs generates strong cross-period d–d orbital coupling to drive directional interfacial charge transfer, synchronously optimizing HER and OER kinetics.^10,27,28^ Compared with mono-, bi- and ternary alloys, quinary multi-d HEAs supply abundant undercoordinated active sites and faster charge transport *via* multi-element synergies, while the sluggish diffusion effect of high-entropy lattices suppresses metal dissolution, reconciling catalytic activity and long-term stability.^21,27^ This design concept promises to overcome the core drawbacks of conventional noble catalysts: exorbitant cost and rapid long-term deactivation. Multi-d-orbital HEA catalytic systems leverage the distinct valence electronic structures of 3d, 4d and 5d transition metals. Hybridization and coupling of diverse d-orbitals enable precise electronic structure regulation, which simultaneously boosts OER activity and cycling durability.^10,19^ Multi-d synergies enhance catalytic performance through multiple pathways: energy level matching and orbital overlap between cross-period metal d-orbitals, collaborative tuning of surface electron density by multivalent metal species, and lattice stabilization derived from multi-element co-alloying.^4,14^ Collectively, these factors comprehensively elevate OER catalytic capability. Meanwhile, intrinsic lattice distortion in HEAs generates abundant undercoordinated defects to expose more active sites, which electronically synergizes with multi-d orbital modulation to further amplify catalytic performance.^19,24^ Although synergistic modulation *via* multi-d orbital coupling holds tremendous potential for designing HEA OER electrocatalysts, related investigations are still in the preliminary exploratory phase and face two prominent bottlenecks. First, core mechanisms including cross-period d–d orbital coupling, interfacial charge transfer pathways and the adsorption energy evolution of oxygen intermediates are insufficiently clarified. Second, existing synthetic methods fail to realize atomic-level homogeneous dispersion of multi-d metallic components within HEA matrices, hindering accurate regulation of elemental stoichiometry and micro-lattice structures.^28–31^ This prevents full utilization of multi-orbital synergistic catalysis and constitutes a key technical challenge for this field.

Targeting the longstanding trade-off between high activity and long-term stability in alkaline OER catalysts, this work proposes a controllable fabrication strategy for quinary HEA electrocatalysts. As cross-period multi-d orbital charge transfer effects between 3d transition metals and 5d Ir have been widely reported in previous literature, the present study focuses on stoichiometric and particle-size regulation rather than revealing an unprecedented electronic mechanism. Carbon-supported IrFeCoNiMn high-entropy nanoparticles are synthesized *via* a facile one-pot oil-phase solvothermal method by alloying Fe, Co, Ni, Mn with noble Ir. Systematic investigations are carried out to clarify how metal stoichiometry and CTAC morphological regulator alter crystal phase, microstructure, surface electronic states and OER performance. Combined multi-dimensional characterizations (XRD, TEM, XPS and electrochemistry) quantitatively separate geometric promotion from lattice distortion and electronic enhancement from well-documented multi-d orbital hybridization coupling, and clarify how such dual synergism governs intermediate adsorption energy, interfacial charge transfer efficiency and catalyst medium-term durability. This work delivers a scalable compositional and dimensional optimization route for high-efficiency low-iridium alkaline OER electrocatalysts suitable for laboratory-scale water electrolysis research.

## Experimental

### Materials

Iron(iii) acetylacetonate (C_15_H_24_FeO_6_, 98%), cobalt(ii) acetylacetonate (C_15_H_24_CoO_6_, ≥99%), nickel(ii) acetylacetonate (C_10_H_14_NiO_4_, 95%), manganese(ii) acetylacetonate (MnC_15_H_21_O_6_, ≥97%) and iridium(iii) acetylacetonate (IrC_15_H_21_O_6_, ≥97%) were purchased from Aladdin Reagents (Shanghai) Co., Ltd. Anhydrous ethanol (C_2_H_5_OH, AR), cetyltrimethylammonium chloride (CTAC, C_19_H_42_ClN, AR) and acetic acid (CH_3_COOH, AR) were supplied by Sinopharm Chemical Reagent Co., Ltd. Vulcan XC-72 carbon (industrial grade) was obtained from Cabot Corporation (USA). Oleylamine (OAm, C_18_H_37_N, 80–90%), cyclohexane (C_6_H_14_, AR) and d-glucose (C_6_H_12_O_6_, AR) were acquired from Macklin Biochemical Co., Ltd. Commercial IrO_2_ powder (Premion grade, 99.99% metals basis) was purchased from Alfa Aesar and used as the benchmark OER electrocatalyst. The deionized water in the experiment is ultrapure (18.25 MΩ cm).

### Fabrication of Ir_16_Fe_24_Co_21_Ni_18_Mn_21_/C catalysts

The Ir_16_Fe_24_Co_21_Ni_18_Mn_21_/C catalyst was fabricated *via* a facile one-pot oil-phase synthetic route. Briefly, 100 mg CTAC was added into a two-neck round-bottom flask containing 20 mL OAm, followed by sequential addition of 12.2 mg IrC_15_H_21_O_6_, 8.8 mg C_15_H_24_FeO_6_, 6.4 mg C_10_H_14_NiO_4_, 8.8 mg C_15_H_24_CoO_6_, and 8.9 mg MnC_15_H_21_O_6_. The mixture was ultrasonicated to form a homogeneous orange-yellow solution, then 10 mg conductive carbon black was introduced and ultrasonic treatment was maintained for another 10 min. Under a nitrogen atmosphere, the mixture was heated to 110 °C at a ramping rate of 5 °C min^−1^ and held at this temperature for 1.0 h, followed by further heating to 250 °C for a 5 h isothermal reaction. After the reaction, the black suspension was centrifuged at 7000 rpm for 5 min. The precipitate was repeatedly washed with an ethanol–cyclohexane mixed solvent (volume ratio = 5 : 1) until the supernatant became colourless. The solid product was collected and dried overnight in a vacuum oven at 60 °C to obtain the target Ir_16_Fe_24_Co_21_Ni_18_Mn_21_/C catalyst. For comparison, a reference sample Ir_15_Fe_24_Co_20_Ni_20_Mn_20_/C was prepared under identical synthetic conditions without CTAC addition. Monometallic Ir/C was also synthesised using the same oil-phase procedure. In addition, two more catalysts, Ir_18_Fe_23_Co_18_Ni_19_Mn_21_/C and Ir_14_Fe_24_Co_25_Ni_17_Mn_20_/C, were synthesised by adjusting the feeding amount of IrC_15_H_21_O_6_ (7.5 mg and 12.5 mg) to tune the metal stoichiometry. The stoichiometric ratios of all as-synthesized catalysts were determined by ICP-OES.

### Electrochemical measurements

All electrochemical measurements were implemented on a CHI760 electrochemical workstation with a thermostatted water bath maintained at 30 °C. A standard three-electrode configuration was adopted, including a graphite rod counter electrode, a Hg/HgO reference electrode and a glassy carbon (GC) working electrode. Freshly prepared 1 mol per L KOH aqueous solution served as the electrolyte. All potentials recorded in this work were converted to the reversible hydrogen electrode (RHE) scale *via* the equation:*E*_RHE_ = *E*_Hg/HgO_ + 0.059 pH + 0.098

The working electrode fabrication procedure was as follows: 10 mg of catalyst powder was dispersed in 2 mL isopropanol containing 0.05 wt% Nafion, followed by sonication for over 20 min to form a homogeneous and stable catalytic ink. Subsequently, 10 µL of the ink was evenly drop-cast onto the GC surface, corresponding to a catalyst loading of approximately 0.2548 mg cm^−2^. Commercial IrO_2_ was fabricated into working electrodes following the identical procedure for performance comparison. Prior to formal testing, each electrode was subjected to 100 consecutive CV cycles at a scan rate of 50 mV s^−1^ to activate the catalyst and stabilize the testing system, after which the electrolyte was replaced with fresh solution for subsequent electrochemical characterizations. Electrochemical impedance spectroscopy (EIS) was recorded over a frequency range of 100 kHz to 0.01 Hz at the overpotential corresponding to a current density of 10 mA cm^−2^. Electrochemical double-layer capacitance (*C*_dl_) was acquired from CV curves collected at various scan rates (20, 40, 60, 80 and 100 mV s^−1^) within the non-faradaic potential window of 1.22–1.32 V *vs.* RHE, which was used to calculate the electrochemically active surface area. The *C*_dl_ value was calculated according to the equation:*j* = *νC*_dl_where *j* is the current density and *ν* denotes the scan rate. Cyclic voltammetry (CV, scan rate = 100 mV s^−1^) and galvanostatic chronopotentiometry (*V*–*T*) measurements were performed to evaluate the cycling stability of catalysts.

### Material characterization

Crystalline phases were identified *via* X-ray diffraction (XRD) on a Bruker D8 Advance diffractometer with Cu Kα radiation, scanning 2*θ* from 30° to 80° at 20° min^−1^. Microscopic morphology, lattice fringes and elemental distribution were characterized using a JEOL JEM-2100F FE-TEM at 200 kV, equipped with HRTEM, STEM and EDS for comprehensive microstructure and elemental mapping analysis. Surface elemental compositions and valence states were probed by X-ray photoelectron spectroscopy (XPS, Thermo Scientific ESCALAB 250Xi), with all spectra calibrated to the C 1s peak at 284.8 eV. Bulk metal stoichiometry was quantified by an Agilent 725 ICP-OES, and the elemental ratios were used to calibrate electrochemical results. ICP-OES sample pretreatment: 5 mg catalyst powder was digested in a PTFE-lined autoclave with 5 mL fresh aqua regia (HCl : HNO_3_ = 7 : 3 v/v) at 180 °C for 24 h in a blast oven. After cooling, the digested liquid was diluted to 50 mL with ultrapure water and mixed uniformly for elemental quantification.

## Results and discussion

**Fig. 1:**
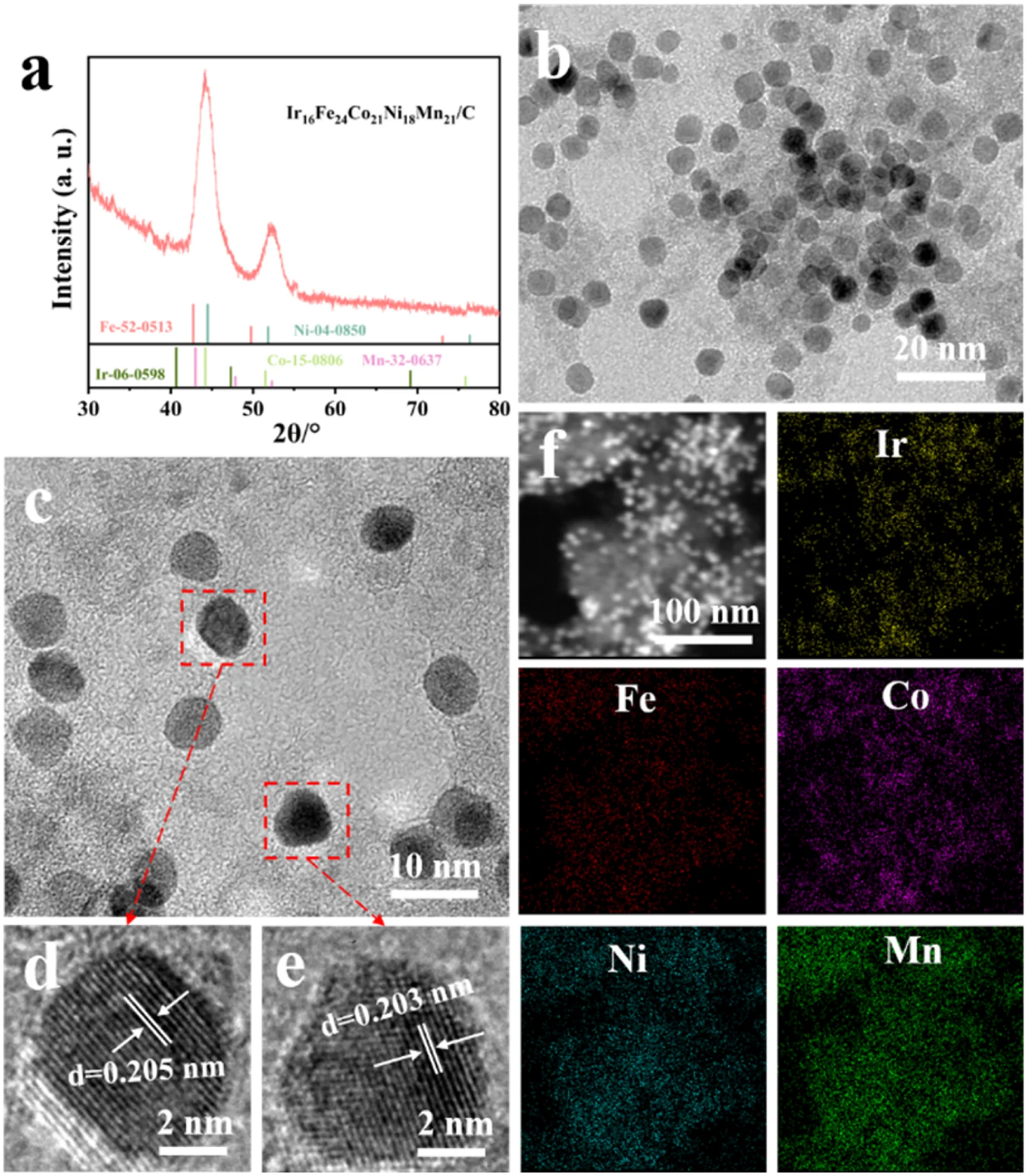
(a) XRD and (b) TEM pattern of Ir_16_Fe_24_Co_21_Ni_18_Mn_21_/C. (c) TEM image; the red square indicates the local region selected for high-resolution TEM analysis. (d and e) High-resolution TEM image taken from the red square area in panel (c). (f) HAADF-STEM image Ir_16_Fe_24_Co_21_Ni_18_Mn_21_/C with elemental mapping images for Ir, Fe, Co, Ni and Mn elements.

In this study, carbon-supported Ir_16_Fe_24_Co_21_Ni_18_Mn_21_ high-entropy alloy (HEA) nanoparticles are fabricated *via* a facile one-pot oil-phase wet chemical route, with the corresponding powder XRD pattern displayed in Fig. 1a. Two distinct diffraction peaks located at 44.23° and 52.32° correspond to the (111) and (200) lattice planes of a single FCC solid-solution phase. Relative to standard diffraction profiles of monometallic Ir, Fe, Co, Ni and Mn, all diffraction maxima of the catalyst exhibit obvious shifts, proving the formation of a homogeneous single-phase alloy accompanied by synchronous lattice contraction and expansion.^32,33^ Severe lattice distortion originates from mismatched atomic radii, disparate electronegativities of the five metallic constituents and intermetallic d–d orbital hybridization. These XRD observations provide solid crystallographic evidence for the successful preparation of the target carbon-supported quinary HEA.^20^ Quantitative ICP-OES analysis (Tables S1 and S2) yields an atomic ratio of Ir : Fe : Co : Ni : Mn = 16 : 24 : 21 : 18 : 21 for Ir_16_Fe_24_Co_21_Ni_18_Mn_21_/C. Each metallic component accounts for 5–35 at%, fully satisfying the compositional threshold for HEAs and further verifying the formation of a high-entropy solid solution from a stoichiometric perspective.^19,22^ Table S3 summarizes calculated thermodynamic parameters: the mixing enthalpy (Δ*H*_mix_) of Ir_16_Fe_24_Co_21_Ni_18_Mn_21_/C falls within the typical HEA range of −20 to 5 kJ mol^−1^; the mixing entropy Δ*S*_mix_ ≥ 1.5*R* (*R* = 8.314 J mol^−1^ K^−1^) and atomic size disparity parameter *δ* ≤ 6.5% also comply with classical empirical criteria for HEA formation, delivering supplementary thermodynamic proof for the obtained HEA electrocatalyst.^10^

Systematic TEM characterizations reveal the micromorphology and crystallographic features of Ir_16_Fe_24_Co_21_Ni_18_Mn_21_/C. The TEM micrograph (Fig. 1b) shows alloy nanoparticles uniformly immobilized on carbon substrates without severe agglomeration, and particle size statistics (Fig. S1) give an average nanoparticle diameter of 5.29 nm. HRTEM images (Fig. 1c–e) present continuous well-defined lattice fringes assigned to the HEA (111) plane, with interplanar spacings measured at 0.205 nm and 0.203 nm. Both values are smaller than the standard (111) interplanar distance of monometallic Ir (0.226 nm), demonstrating prominent lattice distortion within the alloy matrix and consistent with the foregoing XRD results. HAADF-STEM EDS elemental mapping (Fig. 1f) illustrates homogeneous random distribution of Ir, Fe, Co, Ni and Mn without elemental segregation, a characteristic feature derived from the intrinsic high-entropy effect of HEAs. Collectively, complementary structural, morphological and elemental characterizations jointly confirm the successful fabrication of carbon-supported Ir_16_Fe_24_Co_21_Ni_18_Mn_21_ HEA electrocatalysts.

Comparative XRD and TEM characterizations of samples prepared with and without CTAC (Fig. S2a and b) indicate that CTAC acts as an effective surfactant regulating nanoparticle size and dimensional uniformity, while the additive exerts no influence on the inherent FCC crystal phase of HEAs. The correlation between elemental stoichiometry and material microstructure is further explored. Ir_18_Fe_23_Co_18_Ni_19_Mn_21_/C and Ir_14_Fe_24_Co_25_Ni_17_Mn_20_/C display nearly identical morphological and phase features to Ir_16_Fe_24_Co_21_Ni_18_Mn_21_/C (Fig. S2a, S2c and d). Minor stoichiometric adjustments barely induce observable morphological variations, a phenomenon explained by the entropy-stabilization effect unique to multi-d transition metal HEAs. In contrast, the three comparative catalysts without optimized CTAC dosage exhibit poorer particle dispersion and broader particle size distributions, which verifies the critical size homogenization regulation effect of CTAC surfactant (Fig. S3). Three parallel batches of Ir_14_Fe_24_Co_25_Ni_17_Mn_20_/C are synthesized under identical experimental parameters to assess synthetic reproducibility and stoichiometric controllability. ICP-OES data of replicated samples are listed in Table S4, where negligible compositional deviations are detected across parallel trials. This outcome confirms that the one-pot oil-phase synthetic strategy enables precise modulation of metal atomic ratios within HEA nanoparticles, matching conclusions reported in previous literature.^10,33^

**Fig. 2:**
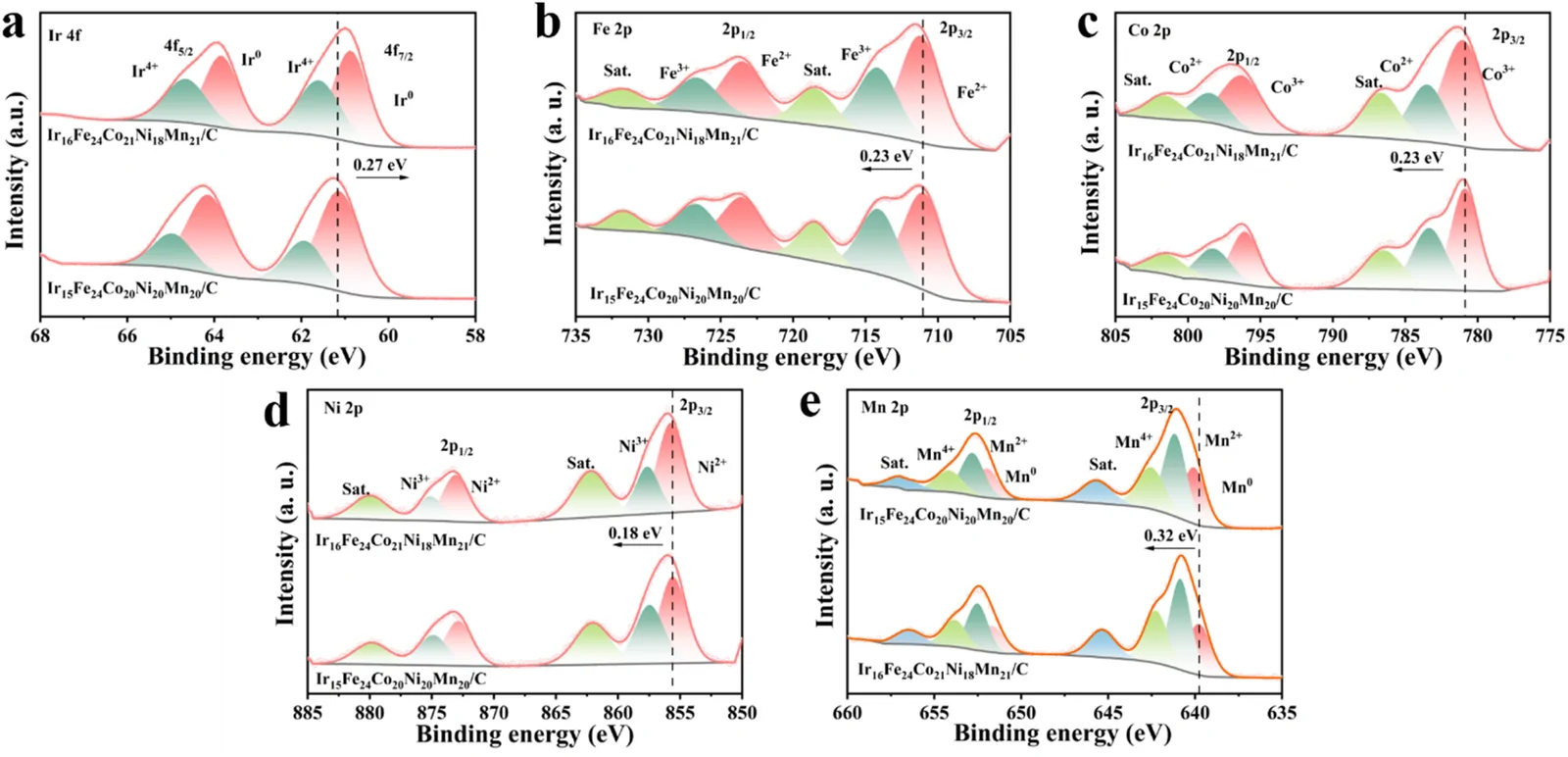
XPS compositional analysis of Ir_16_Fe_24_Co_21_Ni_18_Mn_21_/C. (a) Ir 4f XPS spectrum. (b) Fe 2p XPS spectrum. (c) Co 2p XPS spectrum. (d) Ni 2p XPS spectrum. (e) Mn 2p XPS spectrum.

Surface elemental composition, valence distribution and interfacial electronic interactions of the optimal Ir_16_Fe_24_Co_21_Ni_18_Mn_21_/C HEA catalyst are analysed *via* XPS to reveal how multimetallic valence synergism regulates alkaline OER activity, and the full XPS spectra are compiled in Fig. 2. The high-resolution Ir 4f spectrum (Fig. 2a) splits into two spin–orbit doublets. Peaks at 60.89 eV and 63.85 eV belong to the 4f_7/2_ and 4f_5/2_ orbitals of metallic Ir^0^, whereas signals at 61.62 eV and 64.67 eV are attributed to oxidised Ir^4+^, proving coexisting metallic and high-valent Ir sites on the catalyst surface.^14^ High-resolution Fe 2p, Co 2p and Ni 2p spectra (Fig. 2b–d) each resolve into six peaks consisting of spin–orbit pairs for M^2+^/M^3+^ and satellite signals, confirming that Fe, Co and Ni mainly exist in mixed divalent–trivalent oxide states.^34–36^ The Mn 2p profile (Fig. 2e) is deconvoluted into six primary peaks plus two satellite bands. Peaks at 640.09 eV and 651.97641.17 eV/652.79 eV and 642.58 eV/654.18 eV are assigned to Mn^2+^ and Mn^4+^, respectively.^37,38^ Broad satellite peaks at 645.74 eV and 657.03 eV further verify the coexistence of zero-valent and multi-valent Mn species. Overall XPS results confirm that Ir, Fe, Co, Ni and Mn all feature mixed metallic and oxidised multivalent states on the catalyst surface. This multi-metal multivalent architecture generates strong intermetallic d–d orbital hybridisation, drives directional interfacial charge transfer and remodels the electronic structure of active sites.^23,25^ These electronic modulations balance the adsorption/desorption barriers of key OER oxygenated intermediates and substantially improve intrinsic OER electrocatalytic activity. The clear identification of characteristic valence fingerprints for all five constituent metals also provides solid evidence for the successful synthesis of the quinary Ir_16_Fe_24_Co_21_Ni_18_Mn_21_/C high-entropy alloy.

**Fig. 3:**
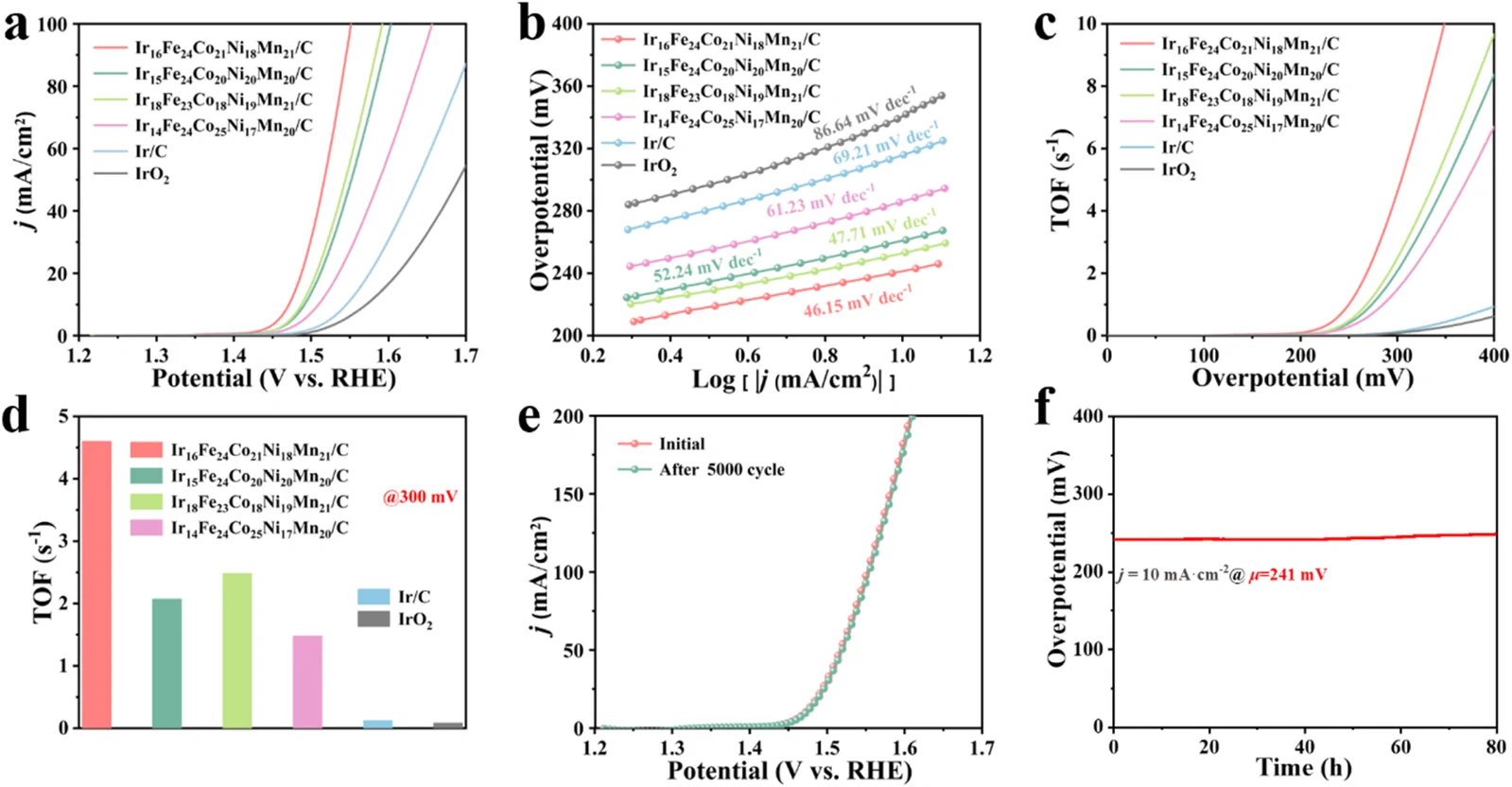
(a) OER polarization curves. (b) Tafel slopes. (c) Relationship between TOF and the measured potentials. (d) TOF values at 300 mV overpotential. (e) Electrochemical ADT test of Ir_16_Fe_24_Co_21_Ni_18_Mn_21_/C in 1 m KOH solution. (f) Long-term stability OER system at 10 mA cm^−2^ for 80 h.

Electrochemical OER performance of as-prepared Ir_16_Fe_24_Co_21_Ni_18_Mn_21_/C HEA is assessed in a standard three-electrode system. The configuration consists of an Ir_16_Fe_24_Co_21_Ni_18_Mn_21_/C-modified glassy carbon working electrode, a Hg/HgO reference electrode and a graphite rod counter electrode, with all measurements conducted in 1.0 mol per L KOH electrolyte. Monometallic Ir/C is prepared as a reference sample, and its structural features are characterised *via* XRD and TEM (Fig. S4). The XRD pattern (Fig. S4a) matches the standard PDF card (Ir-06-0598), confirming pure metallic Ir phase, while the corresponding TEM image (Fig. S4b) shows severe nanoparticle aggregation and broad particle size distribution. Polarisation profiles in Fig. 3a display steep current density growth for Ir_16_Fe_24_Co_21_Ni_18_Mn_21_/C with rising applied potential. At 10 mA cm^−2^, the catalyst delivers an overpotential of only 241 mV, outperforming Ir_15_Fe_24_Co_20_Ni_20_Mn_20_/C (262 mV), Ir_18_Fe_23_Co_18_Ni_19_Mn_21_/C (256 mV), Ir_14_Fe_24_Co_25_Ni_17_Mn_20_/C (278 mV), Ir/C (315 mV), commercial IrO_2_ (340 mV), and numerous reported OER catalysts (Table S6). Tafel slope serves as a kinetic descriptor for electrocatalytic reactions. As presented in Fig. 3b, Ir_16_Fe_24_Co_21_Ni_18_Mn_21_/C delivers an ultralow Tafel slope of 46.15 mV dec^−1^. This value is substantially smaller than all control samples and other electrocatalysts reported in previous studies (Table S6), which reflects the most rapid kinetic behavior for alkaline OER.

TOF is calculated to quantify intrinsic activity and eliminate the influence of catalyst loading, with results summarised in Fig. 3c and d. TOF values of Ir_16_Fe_24_Co_21_Ni_18_Mn_21_/C rise sharply with increased overpotential, reaching 4.603 s^−1^ at 300 mV. This value surpasses Ir_15_Fe_24_Co_20_Ni_20_Mn_20_/C (2.073 s^−1^), Ir_18_Fe_23_Co_18_Ni_19_Mn_21_/C (2.485 s^−1^), Ir_14_Fe_24_Co_25_Ni_17_Mn_20_/C (1.480 s^−1^) and Ir/C (0.126 s^−1^), and is 53.95 times higher than commercial IrO_2_ (0.085 s^−1^). Such superior TOF demonstrates abundant accessible active sites and outstanding intrinsic catalytic activity. Electrochemical impedance spectroscopy (EIS) further reveals charge transport properties (Fig. S5). Ir_16_Fe_24_Co_21_Ni_18_Mn_21_/C delivers a minimal charge transfer resistance (*R*_ct_) of 4.84 Ω, much smaller than all control catalysts, which verifies accelerated interfacial charge migration and favourable OER reaction kinetics. Double-layer capacitance (*C*_dl_) is acquired from CV scans at varied rates within the non-faradaic window of 1.22–1.32 V *vs.* RHE (Fig. S6) to calculate electrochemically active surface area (ECSA). Fig. S7 shows Ir_16_Fe_24_Co_21_Ni_18_Mn_21_/C possesses a *C*_dl_ of 7.81 mF cm^−2^, exceeding all reference materials. The corresponding ECSA reaches 195.25 m^2^ g^−1^ (Table S5), demonstrating that the optimised quinary HEA stoichiometry maximises exposed active sites and accelerates alkaline OER conversion.

Although TOF at 300 mV overpotential reveals the prominent activity advantage of Ir_16_Fe_24_Co_21_Ni_18_Mn_21_/C, this metric relies on a rough approximation that all bulk Ir atoms serve as catalytic active sites, which leads to overestimated active site quantities. Most Ir species are encapsulated within nanoparticle lattices and isolated from alkaline electrolyte, so only surface unsaturated Ir sites participate in OER catalysis, limiting the quantitative accuracy of TOF. To evaluate intrinsic reactivity more rigorously, we normalized polarization currents by ECSA obtained from *C*_dl_ data (Table S5). The full ECSA normalization formula, complete ECSA-normalized LSV profiles for all catalysts, and *j*_ECSA_ values extracted at 300 mV overpotential are provided in Fig. S8. After ECSA normalization, Ir_16_Fe_24_Co_21_Ni_18_Mn_21_/C still exhibits the highest intrinsic current across the entire potential window, confirming that its outstanding OER kinetics originate from multi-d orbital coupling and lattice distortion rather than abundant exposed active sites. To quantitatively separate the geometric (lattice distortion) and electronic (multi-d orbital coupling) contributions, we take commercial IrO_2_ as reference. *R*_ECSA_ = 2.03, *R*_TOF_ = 53.92. Logarithmic decoupling shows lattice distortion accounts for ∼15.1% activity improvement by enriching active sites, while multi-d orbital hybridization dominates the enhancement with ∼84.9% contribution *via* optimized intrinsic single-site activity. This quantitative result solidifies the dual synergistic mechanism.

Long-term cycling stability is another vital criterion for evaluating practical OER electrocatalysts. Accelerated durability testing (ADT) over 5000 CV cycles is performed on Ir_16_Fe_24_Co_21_Ni_18_Mn_21_/C. Polarisation curves before and after show only minor overpotential rise (Fig. 3e), indicating moderate resistance to activity loss. Chronopotentiometry is further conducted at 10 mA cm^−2^ for 80 h (Fig. 3f), and relatively steady potential signals were maintained within the test window (Fig. S9); only a mild 3.26% activity drop was detected after continuous electrolysis. It should be noted this 80 h measurement only evaluates medium-term stability under mild laboratory conditions and cannot represent long-term industrial service performance. Post-stability characterisations are implemented to clarify structural anti-corrosion mechanisms. From the post-OER XRD pattern (Fig. S10a), all characteristic diffraction peaks still correspond to the single FCC solid-solution phase of quinary HEA, with no new oxide impurity peaks observed after 80 h continuous electrolysis. The peak positions barely shift compared with fresh catalyst, demonstrating that the intrinsic lattice structure of Ir_16_Fe_24_Co_21_Ni_18_Mn_21_ high-entropy alloy is well maintained without lattice collapse or phase segregation under alkaline OER oxidative conditions. XRD and TEM (Fig. S10) confirm complete retention of the single FCC phase without nanoparticle aggregation or fragmentation after long-term oxidative electrolysis. We further systematically compared the high-resolution XPS spectra of Ir 4f, Fe 2p, Co 2p, Ni 2p and Mn 2p for fresh and post-OER catalysts (Fig. S11). The peak binding energies, peak splitting shapes and relative area ratios of metallic/oxidized species for each metal exhibit negligible deviations before and after stability testing. This result verifies that the unique charge transfer pathway from 3d metals to 5d Ir is sustained during long-term OER operation, and the multi-valent electronic structure that drives excellent intrinsic activity does not degrade upon electrolysis. Post-test XPS (Fig. S11) presents nearly identical metal valence distributions to fresh catalysts, which preserves the original intermetallic charge-transfer framework. ICP-OES analysis (Fig. S12) only detects trace metal dissolution, as spontaneous electron transfer from 3d transition metals to Ir suppresses noble metal oxidation leaching. Combined characterisation results reveal moderate corrosion resistance and acceptable medium-term catalytic stability of the catalyst within the tested time window, which arises from entropy-stabilised lattice structure and multi-metal synergistic electronic modulation. The material shows potential for short-to-medium term laboratory alkaline electrolysis applications. It should be emphasized that the 10 mA cm^−2^ benchmark current density adopted here is widely used for horizontal performance comparison with published OER catalysts. Systematic long-term durability tests under high industrial-relevant current densities will be carried out in our subsequent research.

Multidimensional characterizations including high-resolution XPS, valence band spectra, EIS, *C*_dl_, ECSA, Tafel plots and TOF calculations are integrated to decode the multi-d orbital synergistic modulation mechanism responsible for the outstanding alkaline OER activity of Ir_16_Fe_24_Co_21_Ni_18_Mn_21_/C. Systematic comparative analyses are performed against five reference catalysts: CTAC-free Ir_15_Fe_24_Co_20_Ni_20_Mn_20_/C, two stoichiometrically adjusted HEAs (Ir_18_Fe_23_Co_18_Ni_19_Mn_21_/C, Ir_14_Fe_24_Co_25_Ni_17_Mn_20_/C), monometallic Ir/C and commercial IrO_2_. High-resolution XPS spectra (Fig. 2) reveal coexisting metallic Ir^0^ and oxidized Ir^4+^ on the catalyst surface, alongside mixed M^2+^/M^3+^ states for Fe, Co, Ni and tri-valent Mn (Mn^0^, Mn^2+^, Mn^4+^). This multi-metal multivalent structure creates favorable conditions for interfacial charge redistribution. Relative to the larger-sized CTAC-free Ir_15_Fe_24_Co_20_Ni_20_Mn_20_/C, positive shifts of 0.23 eV (Fe^2+^ 2p), 0.23 eV (Co^2+^ 2p), 0.18 eV (Ni^2+^ 2p) and 0.32 eV (Mn^0^ 2p) are observed for the target catalyst, accompanied by a 0.27 eV negative shift of Ir^0^ 4f peaks. Such peak displacements confirm that CTAC-induced particle refinement drives spontaneous electron transfer from 3d transition metals to 5d Ir, forming intensive cross-period d–d orbital hybridization and reorganizing the overall electronic structure. This electronic reconstruction balances adsorption affinity toward OER intermediates (*OH, *O, *OOH), resolving the drawbacks of overly strong intermediate binding on pure 3d metals and insufficient adsorption on monometallic Ir.^39,40^ Valence band spectra (Fig. S13) quantify the d-band centre position of all catalysts. The d-band centre of Ir_16_Fe_24_Co_21_Ni_18_Mn_21_/C shifts negatively to −4.155 eV, far lower than the −3.018 eV recorded for commercial IrO_2_ and all other reference samples. This downshift weakens excessive orbital overlap between metal d-orbitals and oxygen intermediates, precisely tuning adsorption/desorption barriers in the four-electron OER pathway: moderate weakening of *O binding and strengthened adsorption of *OH collectively lower the energy barrier of the rate-determining step.^41,42^ EIS results (Fig. S5) support the accelerated charge transfer originating from d–d orbital coupling, as Ir_16_Fe_24_Co_21_Ni_18_Mn_21_/C exhibits an ultralow Rct of 4.84 Ω, much smaller than all control samples. CV-derived *C*_dl_ and ECSA data (Fig. S6, S7 and Table S5) further record a high *C*_dl_ of 7.81 mF cm^−2^ and an ECSA of 195.25 m^2^ g^−1^ for the optimized catalyst. Abundant undercoordinated defects generated by intrinsic lattice distortion and uniform small nanoparticles greatly enrich exposed active sites to boost catalytic activity.

Kinetic data further verify the superiority of multi-d orbital synergy. The catalyst delivers the minimum Tafel slope (46.15 mV dec^−1^) among all tested materials, indicative of rapid elementary reaction steps. At 300 mV overpotential, its TOF reaches 4.603 s^−1^, 2.22 times that of CTAC-free Ir_15_Fe_24_Co_20_Ni_20_Mn_20_/C, 36.53 times that of Ir/C and 53.95 times that of commercial IrO_2_, quantitatively proving superior intrinsic activity. Long-term stability tests validate the dual stabilizing effects of electronic orbital regulation and high-entropy alloying. Polarization curves remain nearly unchanged after 5000 CV cycles, and steady potential output is maintained over 80 h of galvanostatic electrolysis at 10 mA cm^−2^. Post-stability characterizations confirm intact FCC crystal frameworks and uniform nanoparticle morphology *via* XRD and TEM; XPS detects negligible shifts in metal valence distributions, and ICP-OES only records trace metal dissolution. Sustained electron donation from 3d metals suppresses oxidative leaching of noble Ir, while the sluggish diffusion effect of HEAs further restrains metal atom migration. Overall, the synergistic coupling of lattice distortion and multi-d orbital hybridization endows Ir_16_Fe_24_Co_21_Ni_18_Mn_21_/C with lower overpotential, faster reaction kinetics and outstanding long-term durability over all reference electrocatalysts.

## Conclusions

In summary, carbon-supported quinary Ir_16_Fe_24_Co_21_Ni_18_Mn_21_ high-entropy alloy nanoparticles with monophasic FCC solid-solution structure are synthesized *via* a straightforward one-pot oil-phase route, with CTAC introduced as a morphology-controlling surfactant. Joint XRD, TEM and ICP-OES characterizations confirm uniform atomic mixing of Ir, Fe, Co, Ni and Mn, while calculated thermodynamic parameters comply with classic high-entropy formation rules. CTAC effectively reduces particle size and enhances dispersion without disrupting the intrinsic FCC crystal framework. XPS and valence band analyses confirm spontaneous electron migration from 3d transition metals to 5d Ir, which generates intensive intermetallic d–d orbital coupling and shifts the d-band centre to −4.155 eV. Such electronic reconstruction balances the adsorption/desorption strength of key OER intermediates. Electrochemical evaluations reveal abundant accessible active sites and rapid interfacial charge transport for the optimized catalyst. It achieves a low overpotential of 241 mV at 10 mA cm^−2^ alongside a Tafel slope of 46.15 mV dec^−1^, and exhibits a 53.95-fold higher TOF (300 mV overpotential) relative to commercial IrO_2_, surpassing all reference electrocatalysts. Only minor structural degradation, slight valence shift and trivial metal leaching are observed after 5000 cyclic voltammetry cycles and 80 h continuous galvanostatic electrolysis at 10 mA cm^−2^, demonstrating acceptable medium-term stability under the laboratory test conditions. This study focuses on compositional screening and particle-size regulation of IrFeCoNiMn alloys. Tuning elemental stoichiometry and particle dimensions enables quantitative separation of geometric gains from lattice distortion and electronic enhancements *via* multi-d orbital hybridization. The developed stoichiometry- and size-controllable synthetic route offers a practical optimization strategy for low-Ir alkaline OER catalysts, with no novel fundamental catalytic mechanism proposed herein. Future work will focus on evaluating the long-term catalytic durability of this catalyst under high industrial current densities to further explore its practical electrolysis application prospects.

## Author contributions

Xiaolong Ma: conceptualization, methodology, supervision, resources, funding acquisition, writing – original draft, writing – review and editing. Yifan Huang: investigation, methodology. Qin Wang: investigation. Xinger Wang: investigation. Xin Liang: investigation. Gang Hu: resources, supervision. Biao Qin: resources, supervision, writing – review and editing.

## Conflicts of interest

There are no conflicts to declare.

## Supplementary Material

RA-016-D6RA06296B-s001

## Data Availability

The data supporting this article have been included as part of the supplementary information (SI). Supplementary information is available. See DOI: https://doi.org/10.1039/d6ra06296b.

## References

[cit1] Canan O., Bilgen S. Z., Nesrin O. (2025). Energies.

[cit2] Peng P., Shehabi A. (2023). Nat. Sustain..

[cit3] Soleimani L., Refahi A., Amani M. (2026). J. Therm. Anal. Calorim..

[cit4] Chowdhury N., Calais M., Shafiullah G. (2026). J. Energy Storage.

[cit5] Lv M., Qin R., Chen J., Yang K., Lu J., Lei W., Yu Y. (2026). Adv. Sci..

[cit6] Cao Y., Xiang H., Siddique F., Servin-Quintero R. I., Yan Y., Nakum D. K., Wu H., Jie X. M. (2026). Renewable Sustainable Energy Rev..

[cit7] Chen H., Ma Y., Wu Q., Xu H., Zhang L., Wang X., Lyu X., Chen J., Jia Y. (2025). Coord. Chem. Rev..

[cit8] Wu H., Huang Q., Shi Y., Chang J., Lu S. (2023). Nano Res..

[cit9] Zhang F., Niu S., Zhao Y., Li C., Li Z., Lyu S., Hou Y., Baek J. B. (2025). Angew. Chem., Int. Ed..

[cit10] Ma X., Zhou Y., Zhang S., Lei W., Zhao Y., Shan C. (2025). Small.

[cit11] Wang C., Zhang Q., Yan B., You B., Zheng J., Feng L., Zhang C., Jiang S., Chen W., He S. (2023). Nano-Micro Lett..

[cit12] Gui T., Wang Y., Li J., Qiao Z., Wu Z., Sheng Y., Zhuo L., Wu Y., Shakir I., Wu X. (2026). Adv. Energy Mater..

[cit13] Blaseio S., Reetz T., Sözen H. İ., Dosche C., Schneemann C., Sievers G., Rohloff M., Schlickum U., Klüner T., Oezaslan M. (2026). J. Mater. Chem. A.

[cit14] Zhu H., Zhu Z., Meng Y., Feng L., Du B., Zeng D., Zhang W., Gao Q. (2026). New J. Chem..

[cit15] Ma X., Huang J., Ran M., Yang Y., Qin B. (2026). J. Electroanal. Chem..

[cit16] Ramakrishnan S., Vijayapradeep S., Selvaraj S. C., Huang J., Karthikeyan S., Gutru R., Logeshwaran N., Miyazaki T., Mamlouk M., Yoo D. J. (2024). Carbon.

[cit17] Tao J., Fang B., Fang Z., Lin G., Ji Z., Gao C., Gao R., Qiu H. (2025). Angew. Chem., Int. Ed..

[cit18] Ma X., Zhang S., Zhou Y., Lei W., Zhao P., Zhao Y., Luo W. (2024). ChemNanoMat.

[cit19] George E. P., Raabe D., Ritchie R. O. (2019). Nat. Rev. Mater..

[cit20] Zhang B., Mu Q., Pei Y., Hu S., Liu S., Sun T., Gao G. (2025). Nano-Micro Lett..

[cit21] Omelianovych O., Nguyen C. L. T., Szaniel A., Nguyen V.-T., Larina L. L., Choi H.-S. (2026). J. Mater. Chem. A.

[cit22] He J., Hsiao Y.-C., Wu C.-Y., Xia Y., Yang T.-H. (2026). Chem. Rev..

[cit23] Han J., Zhang W., Liu K., Zheng H., Li Y., Luo L., Gong S., Jia Y., Liang X. (2025). Appl. Surf. Sci..

[cit24] Cao X., Lin B.-L. (2026). Nano Res. Energy.

[cit25] Zhao P., Hao X., Pi H., Qi Y., Zhang B., Lei C., Wang J., Zhou N., Chen X., Kan D. (2025). Sci. Adv..

[cit26] Wentao W., Qu Y., Li D., Zhang A., Yan H., Feng Z., Yao W. (2024). Int. J. Hydrogen Energy.

[cit27] Mei Y., Feng Y., Zhang C., Zhang Y., Qi Q., Hu J. (2022). ACS Catal..

[cit28] Zhang Q., Wang Z., Wang L., Wei Z., Zhuo L., Liu Y., Shakir I., Hu G., Liu X. (2025). Nano Res. Energy.

[cit29] Batchelor T. A., Pedersen J. K., Winther S. H., Castelli I. E., Jacobsen K. W., Rossmeisl J. (2019). Joule.

[cit30] Asghari Alamdari A., Jahangiri H., Yagci M. B., Igarashi K., Matsumoto H., Motallebzadeh A., Unal U. (2024). ACS Appl. Energy Mater..

[cit31] Li A., Qureshi N., Maheshwari V. (2025). Nanoscale.

[cit32] Ma X., Zhang S., Zhou Y., Lei W., Zhai Y., Zhao Y., Shan C. (2024). J. Mater. Chem. A.

[cit33] Li H., Han Y., Zhao H., Qi W., Zhang D., Yu Y., Cai W., Li S., Lai J., Huang B. (2020). Nat. Commun..

[cit34] Bagus P. S., Nelin C. J., Brundle C., Crist B. V., Lahiri N., Rosso K. M. (2021). Comments Inorg. Chem..

[cit35] Rattigan E., Jensen S., Sun Z., Nino M. A., Parreiras S. O., Martin-Fuentes C., Martín Romano J. C., Écija D., Escudero C., Villar-Garcia I. J. (2023). J. Phys. Chem. C.

[cit36] Bagus P. S., Nelin C. J., Brundle C. R., Crist B. V., Ilton E. S., Lahiri N., Rosso K. M. (2022). Inorg. Chem..

[cit37] Ilton E. S., Post J. E., Heaney P. J., Ling F. T., Kerisit S. N. (2016). Appl. Surf. Sci..

[cit38] Wu Z., Li X., Li H., Zhang G. (2021). J. Environ. Chem. Eng..

[cit39] Wang G., Li X., Wang J., Yan H., Zhang D., Tian C., Zhang H., Jiao Y. (2025). J. Colloid Interface Sci..

[cit40] Kwon J., Sun S., Choi S., Lee K., Jo S., Park K., Kim Y. K., Park H. B., Park H. Y., Jang J. H. (2023). Adv. Mater..

[cit41] Ma X., Hu S., Wang J., Lv G., Hu Y., Xiao Y., Hu G., Qin B. (2026). New J. Chem..

[cit42] Upadhyay S. N., Joshi H., Sharma N., Pakhira S. (2026). Phys. Chem. Chem. Phys..

