# Supplementary material for: Multi-d orbital and lattice distortion synergism boosting alkaline OER activity of carbon-supported IrFeCoNiMn high-entropy nanoparticles
Source: RSC Adv. 2026 Aug 21;16(41):49198–205. doi: 10.1039/d6ra06296b (PMC13494828; doi:10.1039/d6ra06296b)
Supplement: Supplementary file 1 [file RA-016-D6RA06296B-s001.pdf]

Supplementary Information (SI) for RSC Advances.
This journal is © The Royal Society of Chemistry 2026

Supplementary Information

Multi-d orbital and lattice distortion synergism boosting alkaline OER activity of carbon-supported IrFeCoNiMn high-entropy nanoparticles

Xiaolong Ma*, Yifan Huang, Qin Wang, Xinger Wang, Xin Liang, Gang Hu and Biao Qin*

School of Chemistry and Chemical Engineering, Zunyi Normal University, Zunyi, Guizhou 563006, China;

*Corresponding Author.

E-mail address: shawnma1990@163.com

Calculation of the configurational ΔS_{mix}, ΔH_{mix}, and ΔG_{mix}

The configuration entropy ((ΔS_{mix}) of multi-element alloys increases with the number of different kinds of cations[1]. The calculation of ΔS_{mix} can be performed by using the equation:

$$\Delta S_{mix} = - R\sum C_i InC_i \tag{1}$$

Where R is the gas constant and C_i is the mole fraction of each cation. According to the calculation of the ΔS_{mix}, alloys can be divided into low ((ΔS_{mix}< 1R), medium (1R ≤ (ΔS_{mix}≤ 1.5R), and HEAs (ΔS_{mix} > 1.5R)[1, 2].

It is worth to note that the formation of a solid solution phase of high entropy alloy requires following conditions: atomic radius difference δ ≤ 6.6%, and the mixing enthalpy -11.6 ≤ΔH_{mix}≤ 3.2 kJ mol^{-1} [3]. The atomic radius difference and mixing enthalpy can be calculated by using following equations:

$$\bar{r} = \sum_{i=1}^{n} C_i r_i \tag{2}$$

$$\delta = \sqrt{\sum iC_i\left(1 - \frac{r_i}{\bar{r}}\right)^2} \tag{3}$$

$$\Omega_{ij} = \Delta H_{ij}^{mix} \tag{4}$$

$$\Delta H_{mix} = \sum_{i=1\ i \neq j}^{n} \Omega_{ij} C_i C_j \tag{5}$$

where r is the average atomic radius in the alloy, r_i is the atomic radius of the i_{th} metallic element, ΔH_{mix} is the mixing enthalpy of the binary ij alloy.[1]

Calculation of the turnover frequency (TOF)

The turnover frequency (TOF) was calculated using the following formula:

$$TOF = jA/4Fn \tag{6}$$

where " j " represents the current density at a specific overpotential, " A " stands for the surface area of the electrode, "4" represent the number of moles of electrons

transferred to produce one mole of O_2, " F " represents the Faraday constant (96,485 C mol^{-1}), and " n " represents the number of moles of active sites[4]. The recorded TOFs were based on the assumption that the noble metal atoms present in the samples exhibit catalytic activity.

Calculation of ECSA-normalized specific activity and normalized LSV curves

Raw geometric current density j_{geo} from LSV tests is converted to ECSA-normalized intrinsic current density (j_{ECSA}, mA $cm^{-2}{}_{ECSA}$) via:

$$j_{ESCA}(V) = \frac{j_{geo}(V) \times A_{geo}}{m_{load} \times ESCA \times 10^4} \tag{7}$$

$j_{geo}(V)$: Raw geometric current density (mA cm^{-2}) at arbitrary potential V;

A_{geo}=0.19625 cm^2: Geometric area of 5 mm-diameter glassy carbon electrode;

$m_{load} = 4.998 \times 10^{-5} g$: Total catalyst mass loaded on single working electrode;

ESCA: Electrochemically active surface area (m^2 g^{-1}) of each catalyst (Table S5).

Quantitative decoupling of geometric and electronic contributions

Commercial IrO_2 is set as the baseline to calculate enhancement factors of the optimal $Ir_{16}Fe_{24}Co_{21}Ni_{18}Mn_{21}$/C:

1. Geometric enhancement ratio (ECSA ratio, lattice distortion effect):

$$R_{ECSA} = \frac{ECSA_{Ir_{16}Fe_{24}Co_{21}Ni_{18}Mn_{21}}}{ECSA_{IrO_2}} \tag{8}$$

Lattice distortion nearly doubles exposed active sites.

2. Intrinsic activity enhancement ratio (TOF ratio, multi-d orbital effect):

$$R_{TOF} = \frac{TOF_{Ir_{16}Fe_{24}Co_{21}Ni_{18}Mn_{21}}}{TOF_{IrO_2}} \tag{9}$$

Multi-d orbital hybridization drastically raises single-site intrinsic catalytic capacity.

3. Total activity amplification factor:

$$R_{total} = R_{ECSA} \times R_{TOF} \tag{10}$$

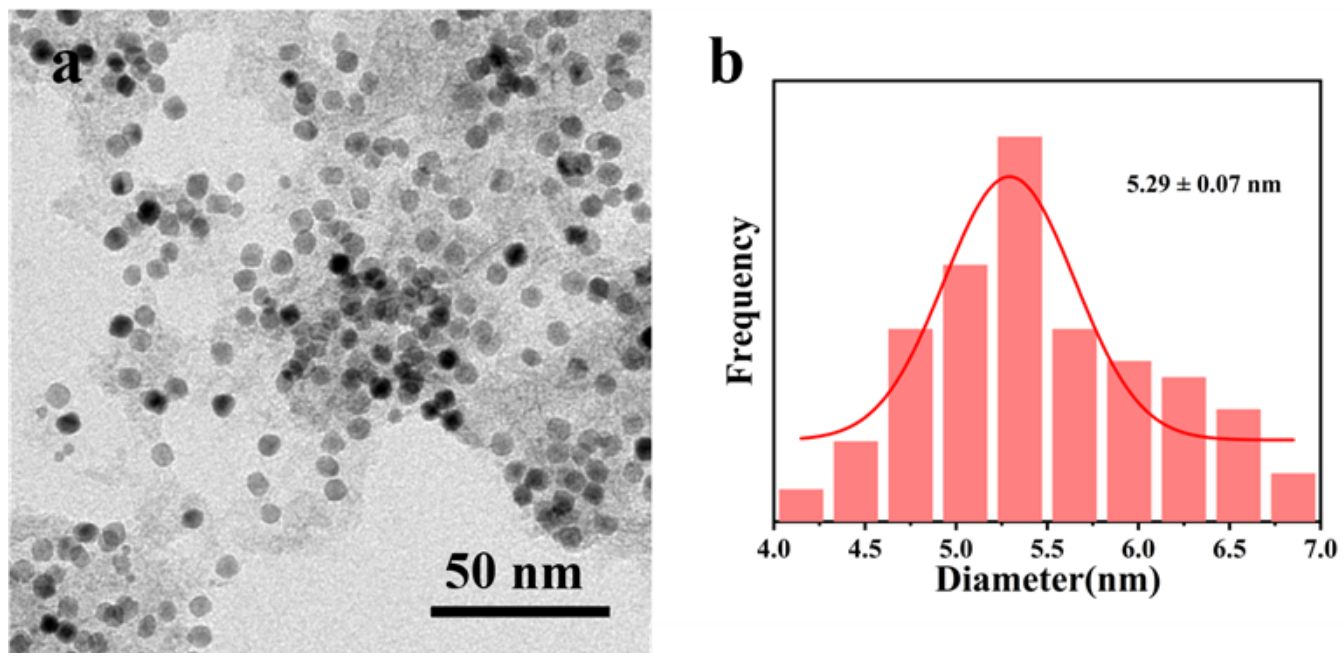

Figure S1. (a) low-magnification TEM imageTEM spectrum, and (b) particle size distribution of $Ir_{16}Fe_{24}Co_{21}Ni_{18}Mn_{21}/C$.

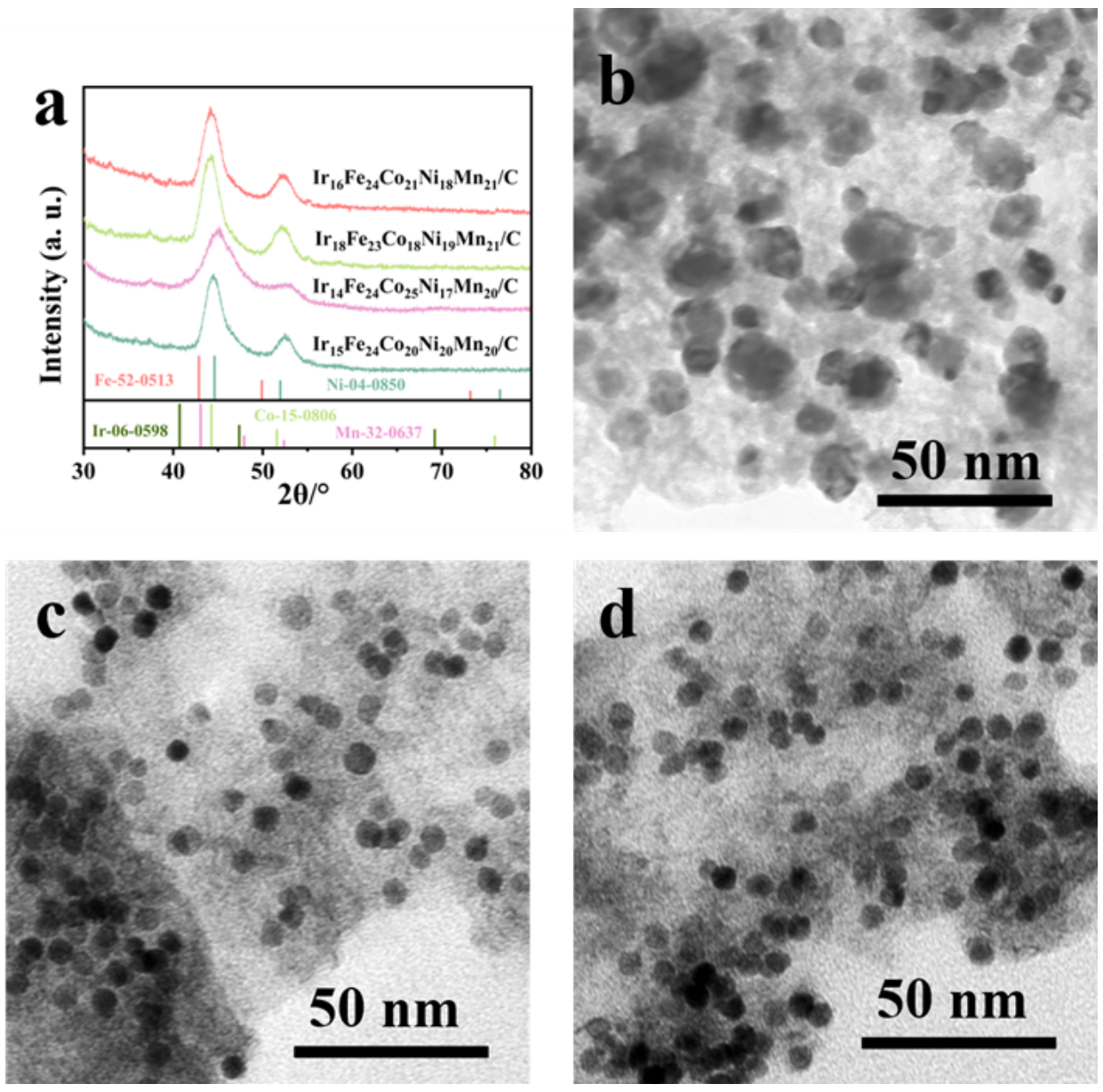

Figure S2. (a) XRD spectrum, and (b–d) TEM image of $Ir_{15}Fe_{24}Co_{20}Ni_{20}Mn_{20}/C$, $Ir_{18}Fe_{23}Co_{18}Ni_{19}Mn_{21}/C$ and $Ir_{14}Fe_{24}Co_{25}Ni_{17}Mn_{20}/C$ catalysts.

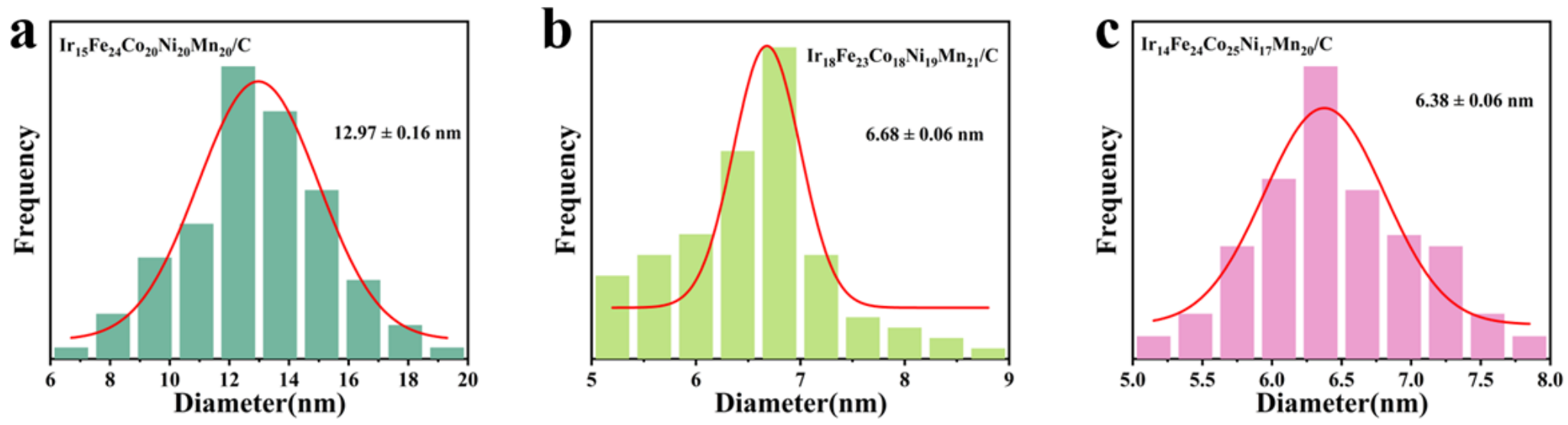

Figure S3. (a-c) particle size distribution of $Ir_{15}Fe_{24}Co_{20}Ni_{20}Mn_{20}/C$, $Ir_{18}Fe_{23}Co_{18}Ni_{19}Mn_{21}/C$ and $Ir_{14}Fe_{24}Co_{25}Ni_{17}Mn_{20}/C$.

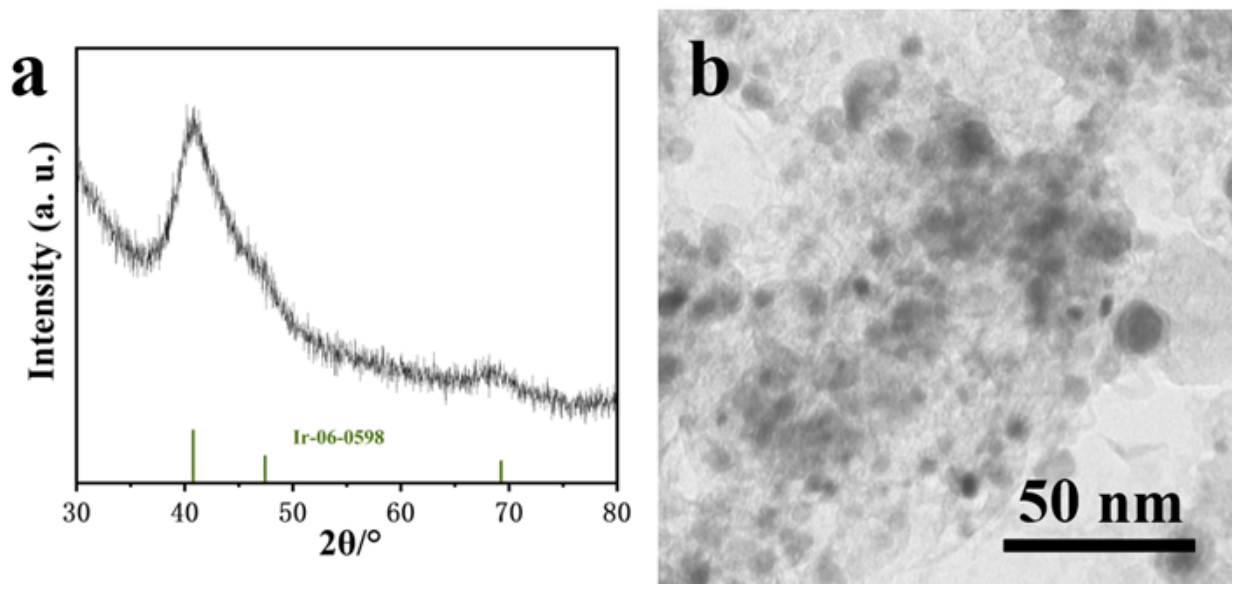

Figure S4. (a) XRD spectrum, and (b) TEM image of Ir/C.

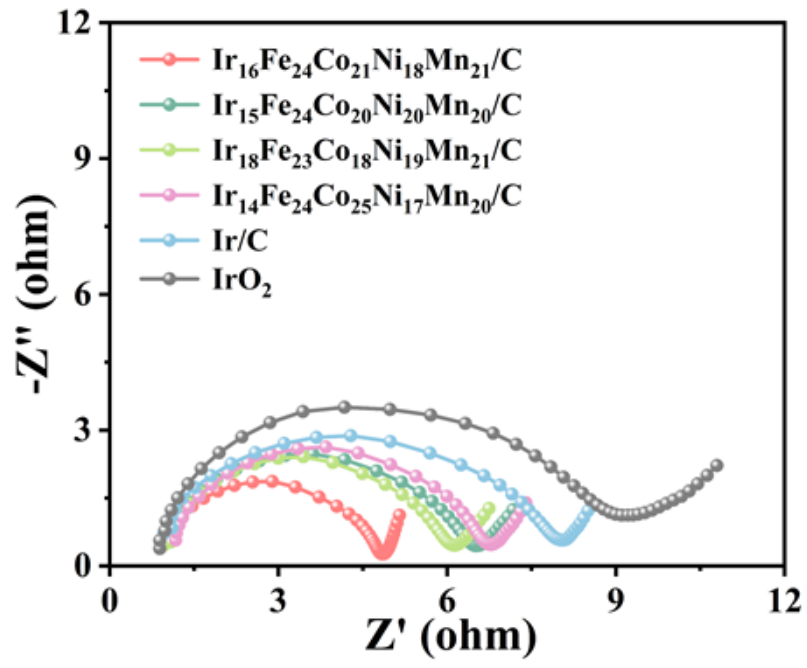

Figure S5. Nyquist plots of catalysts for OER at overpotential of 300 mV.

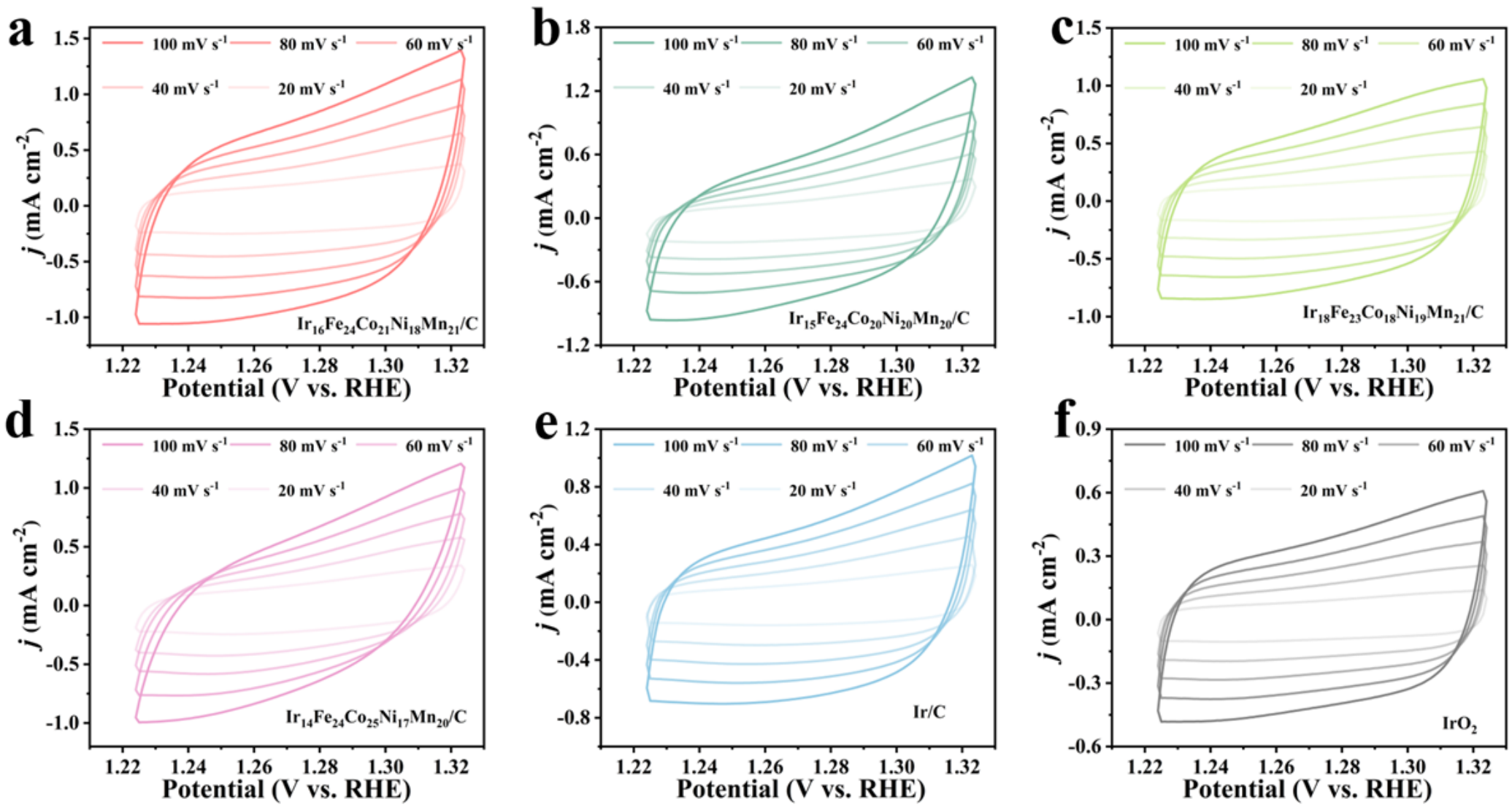

Figure S6. CV curves of (a) $Ir_{16}Fe_{24}Co_{21}Ni_{18}Mn_{21}/C$, (b) $Ir_{15}Fe_{24}Co_{20}Ni_{20}Mn_{20}/C$, (c) $Ir_{18}Fe_{23}Co_{18}Ni_{19}Mn_{21}/C$, (d) $Ir_{14}Fe_{24}Co_{25}Ni_{17}Mn_{20}/C$, (e) Ir/C, and (e) IrO_2/C in the double layer region at scan rates of 20, 40, 60, 80 and 100 mV s^{-1} in 1.0 M KOH.

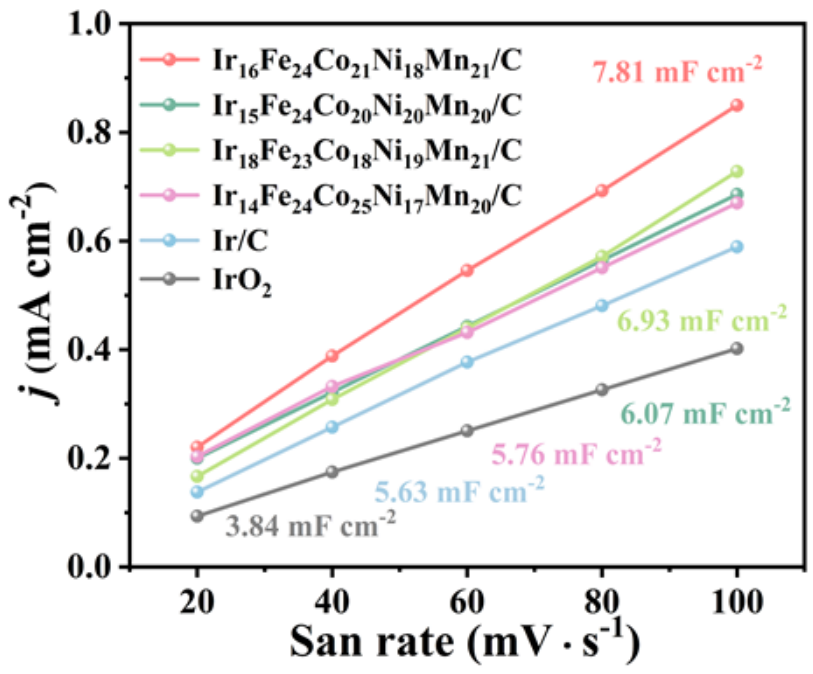

Figure S7. The C_{dl} capacitance of catalysts.

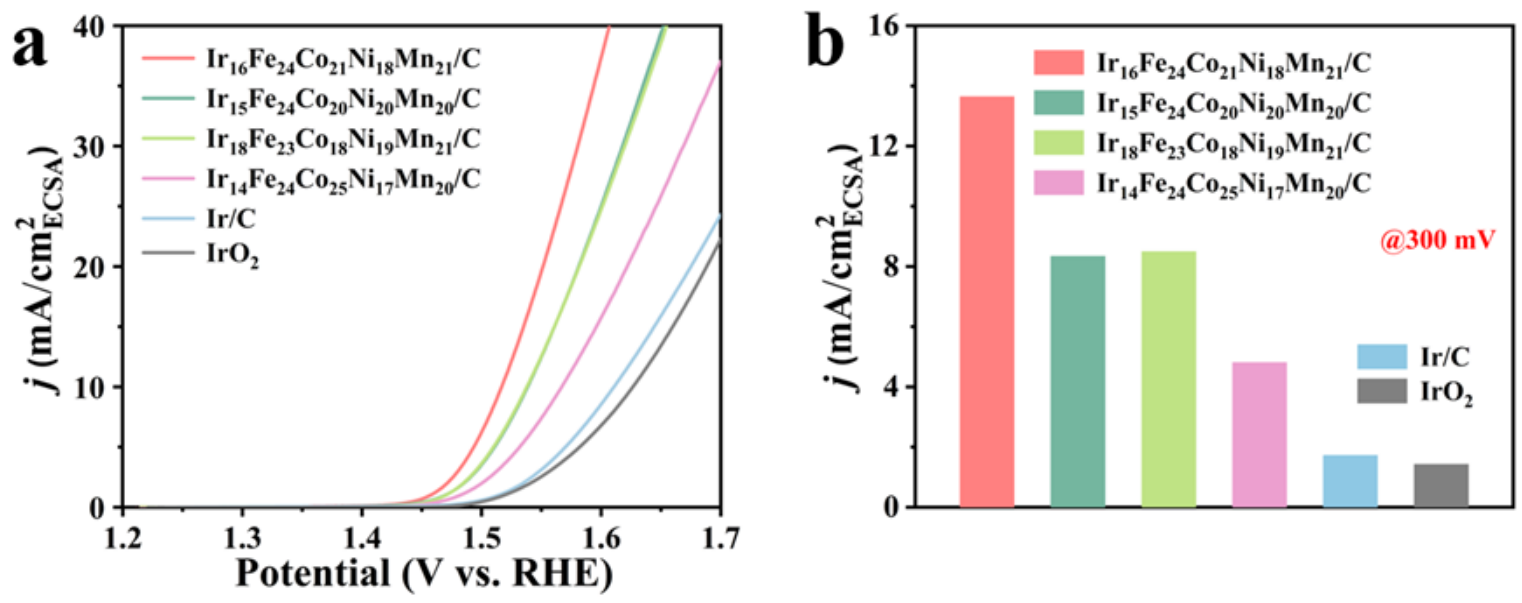

Figure S8. (a) ECSA-normalized LSV polarization curves of all catalysts for OER; (b) Comparison of ECSA-normalized specific activity at an overpotential of 300 mV.

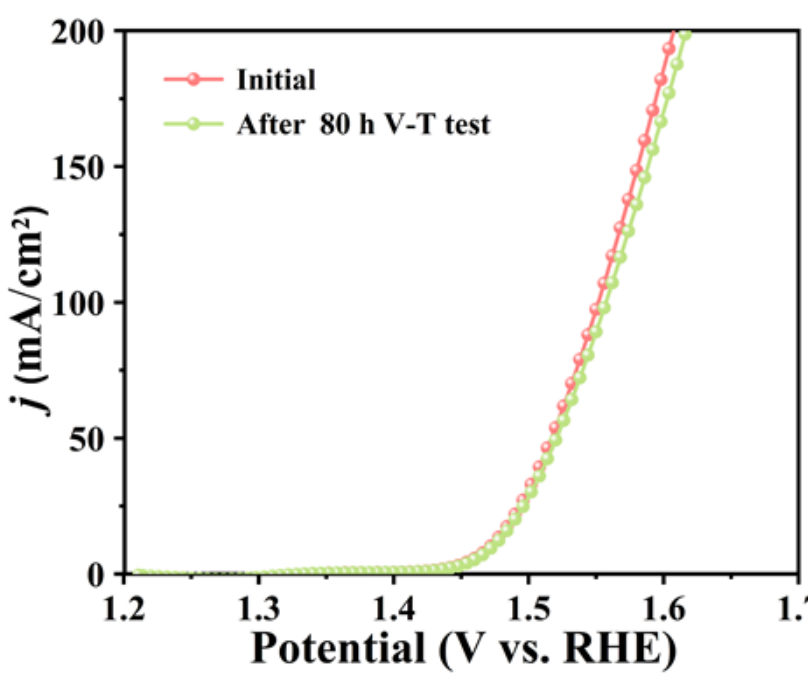

Figure S9. Cyclic voltammetry curves before and after the 80 h V-T stability test.

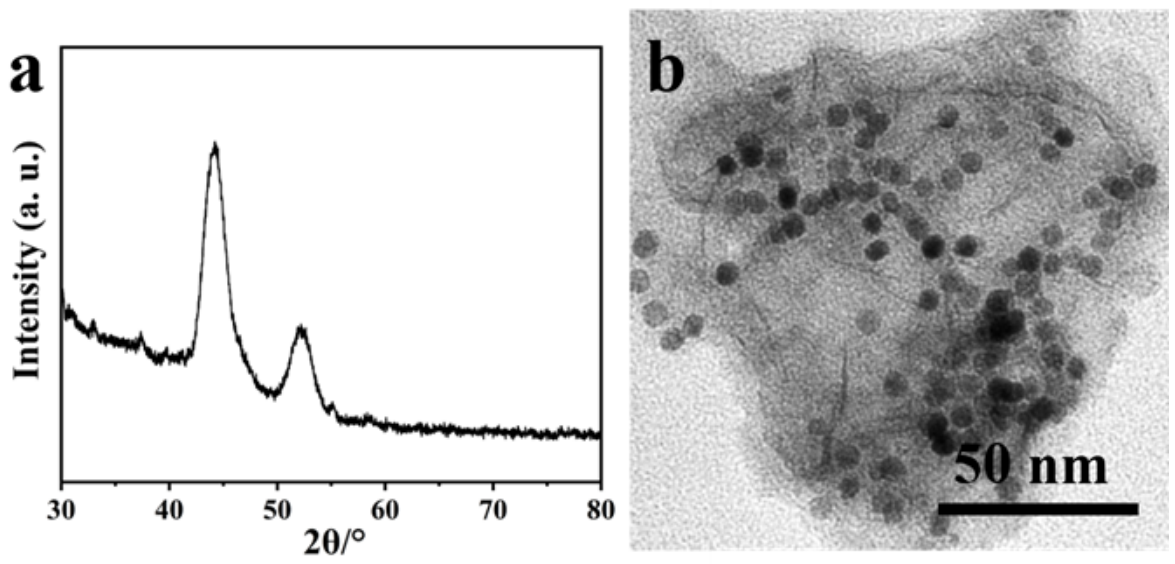

Figure S10. (a) XRD spectrum, and (b) TEM image of $Ir_{16}Fe_{24}Co_{21}Ni_{18}Mn_{21}/C$ catalyst after more than 80 h stability of OER.

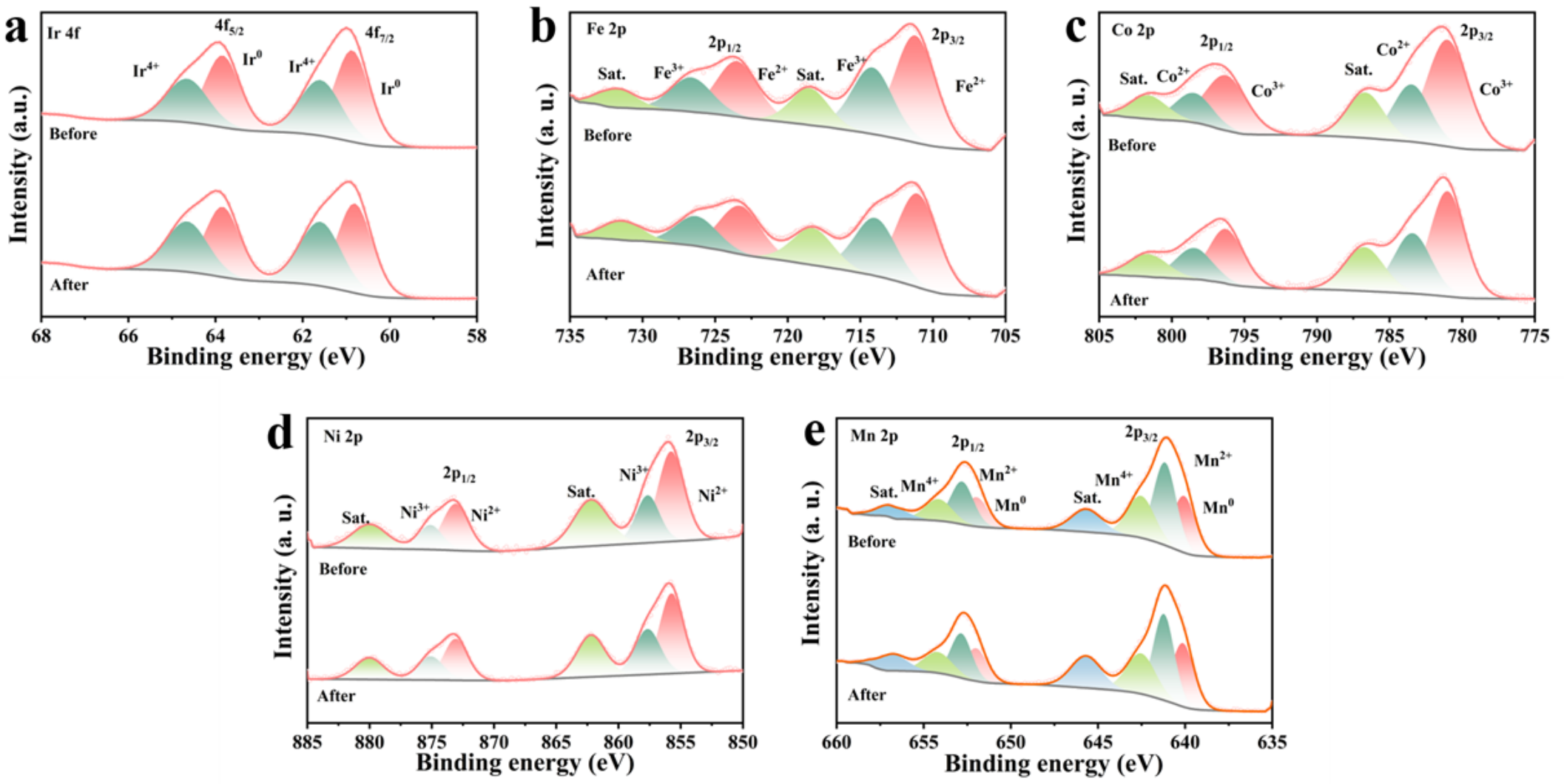

Figure S11. High-resolution XPS spectra of Ir 4f (a), Fe 2p (b), Co 2p (c), Ni 2p (d), and Mn 2p (e) in $Ir_{16}Fe_{24}Co_{21}Ni_{18}Mn_{21}/C$ after more than 80 h stability of OER.

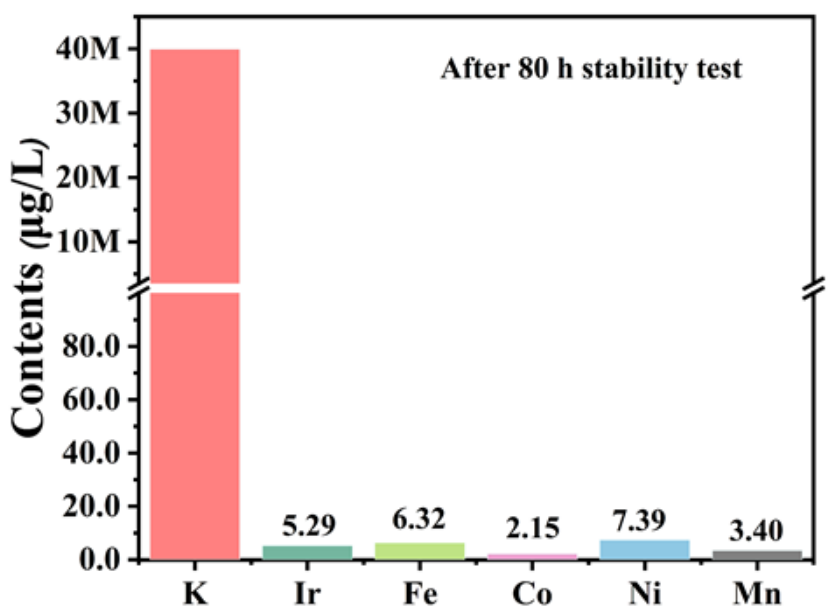

Figure.S12 The ICP-OES results of $Ir_{16}Fe_{24}Co_{21}Ni_{18}Mn_{21}/C$ after 80 h stability test confirm that only trace elements are dissolved.

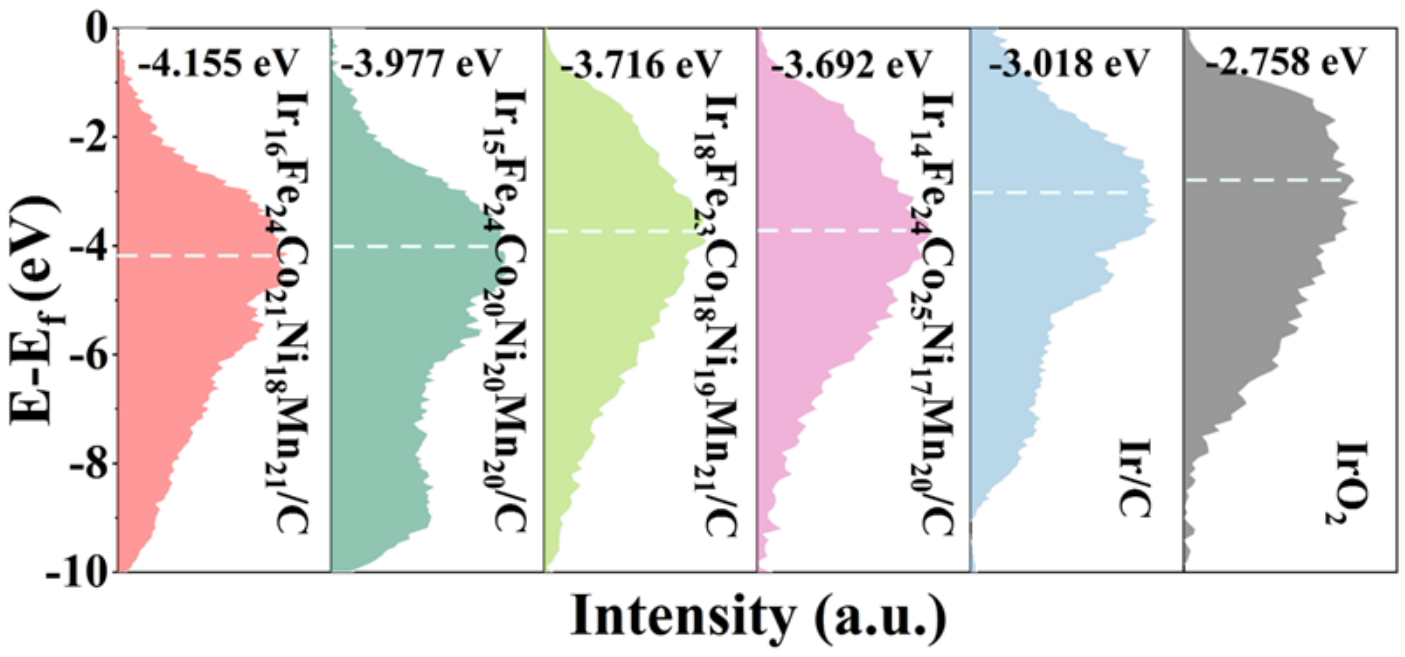

Figure S13 The d-band centers of different catalysts.

Table S1. ICP-OES results of the contents of Ir, Fe, Co, Ni and Mn in HEA/C.

Catalysts	Elements (wt%)				
	Ir	Fe	Co	Ni	Mn
$Ir_{16}Fe_{24}Co_{21}Ni_{18}Mn_{21}/C$	3.03	1.31	1.22	1.03	1.11
$Ir_{15}Fe_{24}Co_{20}Ni_{20}Mn_{20}/C$	3.21	1.51	1.33	1.29	1.24
$Ir_{18}Fe_{23}Co_{18}Ni_{19}Mn_{21}/C$	3.73	1.41	1.14	1.21	1.27
$Ir_{14}Fe_{24}Co_{25}Ni_{17}Mn_{20}/C$	2.46	1.27	1.39	0.97	1.05

Table S2. ICP-OES results of the contents of Ir, Fe, Co, Ni and Mn in HEA/C.

Catalysts	Elements (atomic rate%)				
	Ir	Fe	Co	Ni	M
$Ir_{16}Fe_{24}Co_{21}Ni_{18}Mn_{21}/C$	16.14	24.01	21.19	17.97	20.69
$Ir_{15}Fe_{24}Co_{20}Ni_{20}Mn_{20}/C$	15.06	24.39	20.36	19.83	20.36
$Ir_{18}Fe_{23}Co_{18}Ni_{19}Mn_{21}/C$	18.01	23.43	17.96	19.14	21.46
$Ir_{14}Fe_{24}Co_{25}Ni_{17}Mn_{20}/C$	13.51	23.99	24.89	17.44	20.17

Table S3. The mixing enthalpy (ΔH_{mix}), mixing entropy (ΔS_{mix}), and atomic radius difference of each alloy.

Catalysts	ΔH_{mix} ($kJ\cdot mol^{-1}$)	ΔS_{mix} ($J\cdot K^{-1}\cdot mol^{-2}$)	δ
$Ir_{16}Fe_{24}Co_{21}Ni_{18}Mn_{21}/C$	-9.72	13.30	2.11
$Ir_{15}Fe_{24}Co_{20}Ni_{20}Mn_{20}/C$	-10.31	13.33	2.14
$Ir_{18}Fe_{23}Co_{18}Ni_{19}Mn_{21}/C$	-9.10	13.19	2.09
$Ir_{14}Fe_{24}Co_{25}Ni_{17}Mn_{20}/C$	-9.53	13.29	2.13

Table S4. Results of three parallel ICP-OES tests on $Ir_{16}Fe_{24}Co_{21}Ni_{18}Mn_{21}/C$ catalysts.

Number	Elements (wt%)				
	Ir	Fe	Co	Ni	Mn
1	3.03	1.31	1.22	1.03	1.11
2	2.98	1.34	1.38	0.98	1.18
3	3.02	1.26	1.22	1.02	1.12

Table S5. The double-layer capacitance (C_{dl}), the electrochemically active surface area (ECSA) difference of each catalyst.

Catalysts	C_{dl} (mF cm^{-2})	ECSA (m^2/g)
$Ir_{16}Fe_{24}Co_{21}Ni_{18}Mn_{21}/C$	7.81	195.25
$Ir_{15}Fe_{24}Co_{20}Ni_{20}Mn_{20}/C$	6.07	151.75
$Ir_{18}Fe_{23}Co_{18}Ni_{19}Mn_{21}/C$	6.93	173.25
$Ir_{14}Fe_{24}Co_{25}Ni_{17}Mn_{20}/C$	5.76	144.00
Ir/C	5.63	140.75
IrO_2/C	3.84	96.00

Table S6. Comparison of OER performance of $Ir_{16}Fe_{24}Co_{21}Ni_{18}Mn_{21}/C$ electrocatalyst with recently reported catalysts.

materials	Electrolyte	η_{10}	Tafel slope	Ref.
		(10 mA cm^{-2})	(mV dec^{-1})	
$Ir_{16}Fe_{24}Co_{21}Ni_{18}Mn_{21}/C$	1.0 M KOH	241	46.15	This work
RuO_x/γ-MnO_2	1.0 M KOH	255	84.5	[5]
Ir-NMO	1.0 M KOH	253	89.9	[6]
Ir_1/Co_3O_4	1.0 M KOH	246	58.7	[7]
IrPdCuFeNiCoMo HEA	1.0 M KOH	235	51.0	[8]
PtIrFeNiCoCe	1.0 M KOH	486	/	[9]
FeCoNiCuAl/C	1.0 M KOH	280	/	[10]
FeCoNiPtRu	1.0 M KOH	331	51	[11]
IrFeCoNiMo	1.0 M KOH	249	50.26	[12]
CrFeCoNiRu-RuNi	1.0 M KOH	249	72.34	[13]
ZnNiCoIrFe	1.0 M KOH	250	79.8	[14]
Co-$Ru_{0.55}Ir_{0.45}O_x$	1.0 M KOH	242	41.8	[15]
$Ir_2RuCoCuNi$	1.0 M KOH	264	47	[16]
FeCoNiMnCe		267.5	50.35	[17]

References

1. X. Chang, M. Zeng, K. Liu and L. Fu, *Advanced Materials*, 2020, **32**, 1907226.
2. X. Fa, R. Geng, Z. Zhang, Y. Wang, F. Zheng and S. Yin, *Chemical Engineering Journal*, 2026, 175454. https://doi.org/10.1016/j.cej.2026.175454
3. E. P. George, D. Raabe and R. O. Ritchie, *Nature reviews materials*, 2019, **4**, 515-534.
4. X. Ma, Y. Zhou, S. Zhang, W. Lei, Y. Zhao and C. Shan, *Small*, 2025, **21**, 2411394.
5. T. Pan, W. H. Huang, H. Jie, M. H. Yeh, K. Li, M. Chen, X. Zhou, J. Ge, X. Li and X. Zhao, *Advanced Functional Materials*, 2026, **36**, e17063.
6. J. Zhu, X. Peng, M. Guo, Y. Liu, D. Chao, D. Gao and L. Li, *Angewandte Chemie International Edition*, 2025, **64**, e202516903.
7. Y. Gao, H. Jin, R. Gong, B. Tian, G. Chen, J.-X. Lin, R. Jiang, C. Ji, Y. Zhu and Y. Ding, *Journal of the American Chemical Society*, 2026. https://doi.org/10.1021/jacs.6c03983
8. M. Long, S. Lai, K. Miao, W. Jiang, W. Fan and X. Kang, *Angewandte Chemie International Edition*, 2025, **64**, e202419956.
9. J. H. Cha, S. H. Cho, D. H. Kim, D. Jeon, S. Park, J. W. Jung, I. D. Kim and S. Y. Choi, *Advanced Materials*, 2023, **35**, 2305222.
10. X. Zhu, F. Xue, H. Zhu, W. Huang, H. Yu, S. Peng, W.-D. Liu, R. Yu, J. Zeng and S. Lan, *Nano Research*, 2026. https://doi.org/10.26599/nr.2026.94908613
11. M. Geng, Y. Zhu, J. Guan, R. Zhang, Q. Zou, L. Wang, B. Guo and M. Zhang, *Journal of Alloys and Compounds*, 2024, **1005**, 176180.
12. X. Ma, J. Huang, M. Ran, Y. Yang and B. Qin, *Journal of Electroanalytical Chemistry*, 2026, 120236. https://doi.org/10.1016/j.jelechem.2026.120236
13. Y. Feng, Q. Ma, W. Zhu, K. Harrath, Y. Wei, Y. Wang, Y. Qi, J. Zhang, W. Li and J. Zhu, *Small*, 2026, **22**, e10098.
14. J. Kwon, S. Sun, S. Choi, K. Lee, S. Jo, K. Park, Y. K. Kim, H. B. Park, H. Y. Park and J. H. Jang, *Advanced materials*, 2023, **35**, 2300091.
15. Z. Gan, J. Cao, Z. Chen, P. Qiu, J. Bai, C. Shu and W. Tang, *Nanoscale*, 2025, **17**, 20096-20106.
16. X. Hao, Y. Qi, S. Ding, S. Ma, B. Xu, B. Zhang, Q. Cao and P. Zhao, *Journal of Colloid and Interface Science*, 2025, **683**, 1096-1105.
17. J. Hu, Z. Li, X. Lei, Y. Wang, W. Peng, S. Cui, K. Chen, G. Liu, T. Lang and P. Zhu, *ACS nano*, 2026. https://doi.org/10.1021/acsnano.5c18246
